# Dielectric metalens for miniaturized imaging systems: progress and challenges

**DOI:** 10.1038/s41377-022-00885-7

**Published:** 2022-06-28

**Authors:** Meiyan Pan, Yifei Fu, Mengjie Zheng, Hao Chen, Yujia Zang, Huigao Duan, Qiang Li, Min Qiu, Yueqiang Hu

**Affiliations:** 1 Jihua Laboratory, Foshan, 528200 China; 2 College of Mechanical and Vehicle Engineering, Hunan University, Changsha, 410082 China; 3 Greater Bay Area Institute for Innovation, Hunan University, Guangzhou, 511300 Guangdong Province China; 4 State Key Laboratory of Modern Optical Instrumentation, College of Optical Science and Engineering, Zhejiang University, Hangzhou, 310027 China; 5 Key Laboratory of 3D Micro/Nano Fabrication and Characterization of Zhejiang Province, School of Engineering, Westlake University, 18 Shilongshan Road, Hangzhou, 310024 China; 6 Institute of Advanced Technology, Westlake Institute for Advanced Study, 18 Shilongshan Road, Hangzhou, 310024 China

**Keywords:** Metamaterials, Nanophotonics and plasmonics

## Abstract

Lightweight, miniaturized optical imaging systems are vastly anticipated in these fields of aerospace exploration, industrial vision, consumer electronics, and medical imaging. However, conventional optical techniques are intricate to downscale as refractive lenses mostly rely on phase accumulation. Metalens, composed of subwavelength nanostructures that locally control light waves, offers a disruptive path for small-scale imaging systems. Recent advances in the design and nanofabrication of dielectric metalenses have led to some high-performance practical optical systems. This review outlines the exciting developments in the aforementioned area whilst highlighting the challenges of using dielectric metalenses to replace conventional optics in miniature optical systems. After a brief introduction to the fundamental physics of dielectric metalenses, the progress and challenges in terms of the typical performances are introduced. The supplementary discussion on the common challenges hindering further development is also presented, including the limitations of the conventional design methods, difficulties in scaling up, and device integration. Furthermore, the potential approaches to address the existing challenges are also deliberated.

## Abstract (web summary)

This review outlines the exciting developments in high-performance dielectric metalenses whilst highlighting the challenges of using dielectric metalenses to replace conventional optics in miniature optical systems.

## Graphical abstract

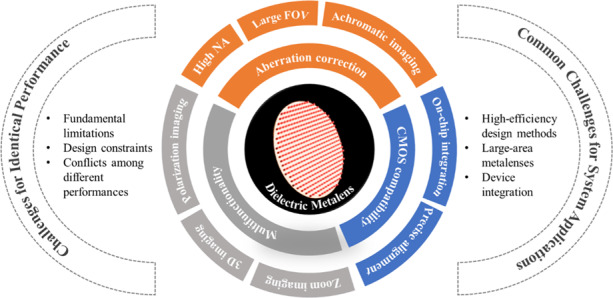

## Introduction

Miniscule and lightweight imaging systems are increasingly desired in consumer electronics, industrial, medical, and automotive markets. The primary component in such systems is the optical lens that focuses light. Therefore, it is critical to develop lenses that are small in size. With the development of freeform lenses or digital surfacing, thinner refractive lenses with lesser weight are achievable. Microlens-arrays in thickness comparable to several wavelengths are also developed with advanced nanofabrication techniques. However, light focusing by conventional lenses relies on the accumulated propagation phase, and it is difficult to downscale further as sufficient accumulated phase is challenging due to the limited refractive indices of natural materials. Moreover, cascading lenses are usually required for high imaging quality, leading to a bulky architecture and challenges in precise alignment. Diffractive lenses, which rely on constructive interference of transmitted light manipulated by spatially arranging “zones”, have been proposed as planar optical lenses^1^. However, they are often impeded by low efficiency, high dispersion, shadowing effect, and integration difficulties^2^. Furthermore, it is difficult for a single diffractive lens to replace multiple refractive lenses.

As an alternative, the metasurface-based flat lens, metalens, could overcome most of the existing challenges^3–8^. Metalens focusing is achieved by abrupt phase change locally imparted by subwavelength structures, namely, meta-atoms. Plasmonic metalenses are firstly demonstrated with metallic nanoantennas^9^, but they suffer from large intrinsic loss. To improve the overall efficiency, all-dielectric metalenses consisting of materials with a high refractive index and low loss are preferred. With the rapid progress in meta-optics, dielectric metalenses have shown subsequent advantages compared to the conventional refractive lenses: (i) corrections for monochromatic and chromatic aberrations are both enabled by a single layer or a few layers of nanostructures, although one needs to manipulate the conflicts among lens parameters (e.g., the trade-off between numerical aperture (NA) and field of view (FOV) for correcting multiple aberrations). (ii) In virtue of a high degree of freedom in shaping the wavefront, multiple functions are simultaneously achievable via a single metalens, opening a new era for building diverse fascinating optical devices. For instance, a single-shot polarization camera is achieved by a metalens paired with an image sensor while the corresponding conventional division of focal plane system consists of beam splitters, polarizers, waveplates, cascading lenses, and multiple detectors. (iii) The fabrication of metalenses is compatible with the complementary metal-oxide-semiconductor (CMOS) fabrication process in the microelectronics industry^2^. Hence, the metalenses have the potential to be directly integrated with the image sensor and the precise alignment of the component elements is enabled by the well-developed techniques in semiconductor foundries. Thus, dielectric metalenses have been explored in imaging and optical information processing, showing the potential to replace the conventional cascading lenses. However, one must address particular challenges for further technological applications of metalens-based integrated systems.

**Fig. 1:**
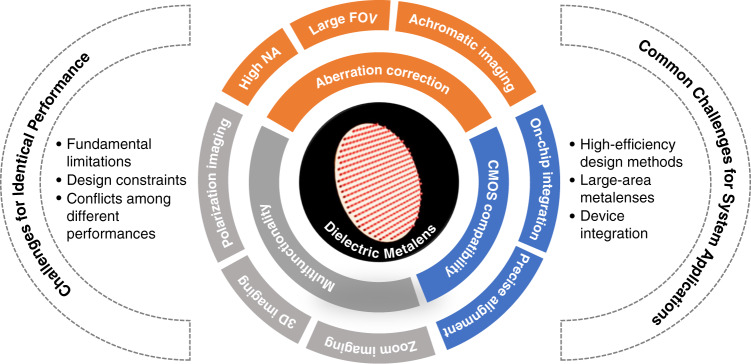
Advantages of dielectric metalenses compared with conventional refractive lenses and the challenges hindering the further development

To encourage the further development of metalens-based compact devices, this review mainly emphasized the progress of dielectric metalenses in compact imaging systems and highlights the challenges obstructing future advancements (Fig. 1). First, a brief introduction to the fundamental physics of dielectric metalenses is presented. The progress and challenges of dielectric metalenses are then introduced in terms of typical performances. Metalenses with a high NA, a large FOV, dispersion engineering capability (for achromatic imaging), and multifunctionality are focused on. The fundamental limitations and design constraints of each performance and conflicts among these performances are discussed further. Subsequently, the existing common challenges hindering the future applications of dielectric metalenses in an integrated system, including limitations of conventional design methods, scaling up of dielectric metalenses, and approaches for integrated devices are highlighted. Finally, we conclude the review with our perspectives on the future work.

## Principle

The design of a metalens typically involves three steps: (i) designing the target phase profile; (ii) constructing a nanostructure-phase library; (iii) matching the target phase with the nanostructure phase. Particularly in step (ii), there are several methods to control the local phase by adjusting the geometrical parameters of meta-atoms, and the nanostructure-phase library is conventionally obtained by parameter sweep.

### Phase profile design

**Fig. 2:**
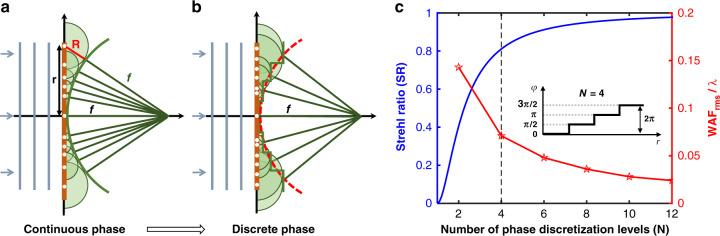
Determination of phase profile for metalens according to Huygens principle. **a** A spherical wavefront is given by the envelope of the secondary spherical waves emitted by sub-sources on a surface with a continuous hyperboloidal phase profile. **b** The meta-atoms impose discrete distribution of phase instead of continuous phase distribution, leading to aberrations related to the wave aberration function (WAF). **c** The effects of the number of phase discretization levels on the normalized WAF_rms_ (WAF_rms_/λ) and the Strehl ratio (SR), which are represented by red and blue lines, respectively. Inset is the case of 4-level phase discretization. The data are extracted from ref. ^11^

According to Huygens principle^10^, each point on a wavefront of a propagating wave is itself the source of spherical wavelets in a homogeneous medium. The wavefront in the next instant conforms to the envelope of these secondary wavelets. As depicted in Fig. 2a, the aberration-free focusing requires a spherical wavefront. Hence, the radius of the secondary wavelet at the radial position *r* should be $R(r) = \sqrt {r^2 + f^2} - f$, where *f* is the focal length of the metalens. Then the phase retardation compared with the reference phase at the center is: $\varphi \left( r \right) = - kR(r)$, where *k* is the wavevector of the light in the medium. As we know, $k = n_b\omega /c = 2\pi n_b/\lambda$, where *n*_*b*_ is the refractive index of the background medium, *c* is the speed of light in the vacuum, $\omega$ is the circular frequency, and $\lambda$ is the vacuum wavelength. Consequently, the normal incidence will focus at a spot after transmitting a surface with a hyperboloidal phase profile:

1 $$\varphi \left( {r,\omega } \right) = \frac{{n_b\omega }}{c}\left( {f - \sqrt {r^2 + f^2} } \right) = \frac{{2\pi n_b}}{\lambda }\left( {f - \sqrt {r^2 + f^2} } \right)$$

In the design of a metalens, the local phase shifts are imposed by nanostructures with finite unit cell size, so discrete phase levels covering a 2*π* phase delay are imparted instead of the continuous phase distribution^11^ (Fig. 2b). The difference between the envelope wavefront of discretized phase profile and the reference spherical wavefront is represented by the wave aberration function (WAF). As the number of phase discretization levels increases, the metalens discrete phases are closer to the continuous counterparts, and the real envelope wavefront is thus more consistent with the ideal one. Consequently, the root mean square of WAF (${\mathrm{WAF}}_{\mathrm{rms}} = \sqrt{ {\langle{\mathrm{WAF}}\rangle}^2 - {\langle{\mathrm{WAF}}^2\rangle}}$ where the brackets represent the mean value^12^) decreases as shown in Fig. 2c (red line). Meanwhile, as a conventional criterion for focusing performance, the Strehl ratio (SR), which is defined as the peak intensity normalized to that of the Airy disk, increases with decreasing ${{{\mathrm{WAF}}}}_{{{{\mathrm{rms}}}}}$^12^. Therefore, the SR increases with the number of phase discretization levels (blue line in Fig. 2c). According to the Marèchal criterion^13^, aberrations are negligible when ${{{\mathrm{WAF}}}}_{{{{\mathrm{rms}}}}}$ is less than λ /14, i.e., ${{{\mathrm{WAF}}}}_{{{{\mathrm{rms}}}}}/\lambda \,< \,0.071$. In this case, diffraction is the dominant factor to limit the imaging quality. As shown in Fig. 2c, the Marèchal criterion is satisfied with a phase discretization into four levels (*N* = 4)^11^, and the corresponding SR is about 0.81. In some cases, for instance, for wide-angle imaging, the envelope may be an aspherical wavefront for the compromise between on-axis and off-axis aberrations (detailed discussion in section “Compromise between high-NA and high FOV”). The ${{{\mathrm{WAF}}}}_{{{{\mathrm{rms}}}}}$ is thus contributed by not only the phase discretization but also the difference between the aspherical wavefront and the reference spherical wavefront. Increasing the number of phase discretization levels (*N*) reduces the WAF_rms_, but a nontrivial fabrication challenge is also posed due to the reduced unit size. Hence, the trade-off between the focusing quality and fabrication should be manipulated based on the application requirements.

Benefitting from the high design freedom, metalens is also popular to focus the customized beams such as vortex beams^14–19^. The desired phase profile, especially those that are too complicated to be presented by an analytical equation, can be extracted from the simulations, or obtained by numerical strategies such as computer-generated holography methods^20,21^.

### Phase modulation mechanisms

**Fig. 3:**
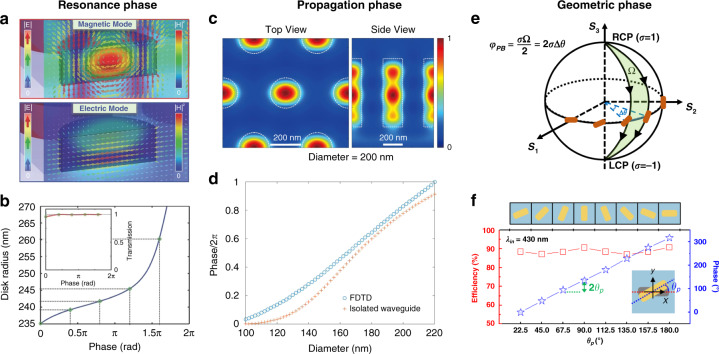
Methods to control abrupt phase imposed by meta-atoms. **a** Field distributions for electric and magnetic dipoles. **b** Amplitude and phase modulation by adjusting the dimensions of resonant meta-atoms. Panels **a** and **b** are reproduced with permission^22^. Copyright 2015, Wiley-VCH. **c** The normalized magnetic field indicating a waveguide mode. Reproduced with permission^46^. Copyright 2018, American Physical Society. **d** Comparison of the phase calculated by FDTD simulation of nanopillar on a glass substrate, and the phase due to propagation in an isolated cylindrical waveguide. Reproduced with permission^35^. Copyright 2016, American Chemical Society. **e** Schematic of the geometric phase in Poincaré sphere. **f** Geometric phase generated by rotating the orientation of anisotropic meta-atoms (blue) and the corresponding conversion efficiency of the incident circular polarization (red). Reproduced with permission^34^. Copyright 2017, American Chemical Society

A metalens is designed by sampling meta-atoms that impose the required local phase for the target. The required 2*π* phase delay imparted by meta-atoms mainly includes phase shift of resonance, propagation phase, and geometric phase, as shown in Fig. 3.

#### Resonant phase

A kind of ultrathin dielectric metalens (thickness $H \ll {\uplambda}$), namely, dielectric Huygens’ metalens is achieved based on resonant-type phase related to excited resonances such as Mie resonance^22–28^ and Fabry–Pérot resonance^29–31^. For instance, strong Mie-type scattering resonances can be excited in nanoparticles made of dielectric material with a high refractive index in the spectral range of interest (Fig. 3a). The excited electric and magnetic dipole moments can be tuned by tailoring the nanodisc’s dimensions, meeting the so-called first Kerker condition, and then allowing a maximum of 2*π* phase shift with near-unity transmission^22^ (Fig. 3b). Using anisotropic nanoantennas introduces the degree of freedom related to polarization manipulation^24^. The birefringent effect also can introduce phase change accompanied by polarization transformation^32,33^. Nevertheless, such resonant type of metalens only operates in a narrow bandwidth. Moreover, the considerable resonance mode coupling between adjacent nanostructures may significantly lead to errors in the case of large phase gradients, degrading the focusing performance^25^.

#### Propagation phase

The dielectric meta-atoms with heights comparable to wavelength (*H*~λ) can be regarded as truncated waveguides^34–37^ (an example is demonstrated in Fig. 3c^35^). In this situation, the phase modulation is based on the propagation phase (also known as the dynamic phase) propagating through the meta-atom:

2 $$\varphi \left( {x,y,\lambda } \right) = \frac{{2\pi }}{\lambda }n_{\rm{eff}}\left( {x,y,\lambda } \right)H$$

where $n_{\rm{eff}}\left( {x,y,\lambda } \right)$ is the local effective refractive index of the meta-atoms. The propagation phase can be adjusted by the physical parameters of each unit cell (the size of the nanostructure, the duty cycle, and lattice periodicity of the unit cell, etc.). For meta-atoms with simple configuration, the $n_{\rm{eff}}$ can be readily computed using single step-index waveguide models. An example is shown in Fig. 3d. For the nanopillars, the phase calculated by an isolated cylindrical waveguide model agrees well with the results obtained via a finite-difference time-domain (FDTD) analysis. The better agreement with larger diameters corresponds to the stronger confinement^35^. Polarization-insensitive metalenses are achieved by isotropic nanostructures^34–36^, while linear-polarization-dependent responses are realized by anisotropic meta-atoms^37^. Similar to resonant-type nanoantennas, phase modulation is achievable by polarization transformation^38^.

#### Geometric phase

For circularly polarized incidence **E**_in_ = [1 ± *i*]^T^, the output electric field ${{{\mathbf{E}}}}_{{{{\mathrm{out}}}}}$ from an anisotropic meta-atom is^7^:

3 $${{{\mathbf{E}}}}_{{{{\mathrm{out}}}}} = C_1e^{i\phi _1}\left( {\begin{array}{*{20}{c}} 1 \\ { \pm i} \end{array}} \right) + C_2e^{i\phi _2}e^{ \mp i2\theta }\left( {\begin{array}{*{20}{c}} 0 \\ { \mp i} \end{array}} \right)$$

where $C_1e^{i\phi _1}$, and $C_2e^{i\phi _2}$ are the transmitted coefficient involving the propagation phase. The first term on the right side of Eq. (3) is the co-polarized component, and the second term is the crossed-polarized component with a phase shift of $2\theta$ (Fig. 3f). Such additional phase shift is originated from the geometric phase, also known as Pancharatnam–Berry (PB) phase^39,40^. PB phase is the extra phase difference caused by paths from one point to another on the Poincaré sphere, so it depends only on the rotation orientations of anisotropic meta-atoms related to points on the Poincaré sphere (Fig. 3e). Hence, broadband phase modulation with $2\pi$ phase coverage can be achieved by rotating identical anisotropic meta-atoms, enabling broadband imaging^41^.

The degree of freedom for phase control can be further extended with the combination of two more types of phase control methods. Considering the phase $\phi _2$ in Eq. (3), the phase profile is achieved by combining propagation and geometric phase by simultaneously adjusting the rotated angle and size of meta-atoms^42–44^. The resonance phase is also combined with the propagation or geometry phase^32^.

## Progress and challenges for typical performances

High-resolution imaging is enabled by a single metalens, which possesses capabilities for monochromatic aberration corrections and dispersive engineering. In addition, multifunctionality can be achieved by the versatile manipulation capabilities of metalenses on electromagnetic fields. By virtue of these high-performance features, metalens is a competitive candidate to replace conventional optical components in integrated or microscale optics for applications such as microscopy, augmented reality (AR), virtual reality (VR), polarization imaging, adaptive zoom, and three-dimensional (3D) imaging. Nevertheless, even with the outstanding performance, multiple challenges, both theoretical and experimental, have to be overcome for the continued development of metalens-based optical applications.

### Monochromatic aberration correction

**Fig. 4:**
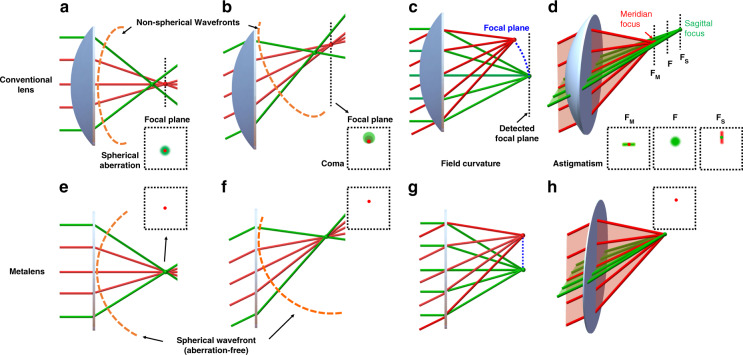
Schematic diagrams for the monochromatic aberrations. Panels **a**–**d** show the aberrations generated by waves passing through conventional spherical lenses: **a** the spherical aberration is generated with the non-spherical wavefront of normal incidence; **b** the coma is generated with the non-spherical wavefront of oblique incidence; **c** the filed curvature occurs when the incident angle-dependent focal spots form a curve plane; **d** astigmatism occurs when the transmitted wavefronts for the meridian beam and sagittal beam correspond to different focal lengths, respectively. Panels **e**–**h** show aberration-free focusing by metalenses that provide ideal spherical wavefronts: **e** spherical aberration-free focusing; **f** coma-free focusing; **g** focusing without field curvature; **h** astigmatism-free focusing. The parallelly normal (oblique) incidence is from an on (off)-axis object at an infinite distance. The dash lines in orange represent the wavefront of light after transmitting the focusing lenses (which could be metalenses and also the conventional counterparts). Insets are the corresponding focal spots

Resolution is a key factor for judging the performance of a lens. According to the diffraction theory of physical optics, the focal spot size of a lens is limited by its NA:

4 $${{{\mathrm{FWHM}}}} = \frac{\lambda }{{2NA}}$$

where FWHM represents the full width of the half maximum of the focal spot size, λ is the incident wavelength, and NA depends on the ambient index (*n*) as well as the collecting angle of marginal ray (*θ*): $NA = n\sin \theta$. With the fixed wavelength, a higher theoretical resolution can be obtained by a lens having a larger NA. However, the actual resolution of conventional lenses is lowered by monochromatic aberration including axis and off-axis aberrations. As aforementioned, spherical wavefronts are required for aberration-free focusing. However, conventional lenses, which are usually manufactured as spherical surfaces, generate non-spherical wavefronts for parallel incidence. Axis aberration, i.e., spherical aberration, occurs when rays are focused on different image planes after passing through the central and marginal regions of the lens (Fig. 4a). Off-axis aberrations include coma, astigmatism, and field curvature. Coma aberration occurs when rays from an off-axis point are focused at different foci in the ideal image plane, forming a comet-like pattern (Fig. 4b); even without spherical and coma aberrations, astigmatism occurs when the convergent point of the meridian beam and the sagittal beam from an off-axis object point cannot be focused at the same position along the propagating direction (Fig. 4d), and the field curvature is the bending of the image plane (Fig. 4c). Therefore, conventional spherical lenses with high NA suffer from great spherical aberration, while the off-axis aberrations are negligible with a large FOV. With the local phase control capability, metalenses could provide spherical wavefronts for both on-axis and off-axis incident light (Fig. 4e, f), so they are widely explored for the high NA and large FOV cases.

#### High-NA metalens

A large NA is desirable for applications requiring small light−matter interaction volumes or large angular collections (e.g., microscopy, and functionalized fiber applications). In conventional optics, the supplementary spherical aberration is usually eliminated by cascading a series of precisely aligned compound lenses or aspherical lenses. In contrast, diffraction-limited imaging with single-piece metalens is achievable.

##### Representative designs and the sampling constraints

Metalenses with hyperbolic phase profiles are originally free of spherical aberration, so it is extendedly explored in high-NA cases. In the work of the Capasso group, a hyperbolic phase profile is implemented with the PB phase controlled by rotating TiO_2_ nanofins for a free-space NA of 0.8^41^. The achievable NA is related to the parameters of the unit cell. According to the Nyquist–Shannon sampling theorem in the spatial domain, the spherical aberration could be prevented when $NA \le \lambda /(2p)$^45^, where *p* is the periodicity of the unit cell. Hence, small periodicities (i.e., high sampling rate) are generally set for large-NA metalens, as listed in Table 1. Note that in Fan’s work^46^, the divergent metalens offers blur images as the periodicity is not small enough for the near-unity NA.

**Table 1:** Examples to show the lattice constraints on NAs of metalenses

Ref.	*λ* (nm)	*p* (nm)	*λ*/(2*p*)	Reported NA	Focusing efficiency	Minimal size; height	Material; phase control
*Science* 2016^41^	405	200	1.01	0.8 (air)	0.86	136.6 nm; 600 nm	TiO_2_; PB phase
	532	325	0.82		0.73		
	660	430	0.77		0.66		
*Nano Lett.* 2017^45^	405	150	1.35	1.1 (water)	0.53	108.7 nm; 600 nm	TiO_2_; PB phase
	532	240	1.108				
*Nano Lett.* 2018^47^	532	220	1.209	0.98 (air)	0.48	36.2 nm; 500 nm	c-Si; PB phase
				1.48 (oil)			
*Phys. Rev. Appl.* 2018^46^	633	416	0.76	0.98 (air)	Divergent	173.75 nm; 695 nm	Si_3_N_4_; Propagation phase
*Nat. Commun.* 2019^48^	700	300	1.167	1.1 (solid)	–	100 nm; 1000 nm	Diamond; Propagation phase

However, coupling between adjacent elements would be inevitable with the decreased unit cells. For PB phase-based metalens, the peak polarization conversion efficiency of the nanofin blue shifts as *p* decreases in a PB phase-based metalens^45^. For waveguide-type metalenses, although the adjacent coupling effect is efficiently reduced by the highly confined electromagnetic field, the range of phase delay is reduced by the limited variation in the lateral dimensions. Hence, meta-atoms with a higher refractive index or higher aspect ratio are preferred to push the NA further. The former, however, is limited by the material, while the latter is restricted by fabrication constraints. In contrast, the easier approach to increase the NA is conventional immersion^45,47^. The highest NA is reported as 1.48 with an oil-immersion c-Si metalens^47^. Moreover, a solid-immersion lens composed of nanoscale diamond pillars is proposed to collect and collimate the emission of an individual nitrogen-vacancy (NV) center^48^.

##### Approaches to relax sampling restriction

**Fig. 5:**
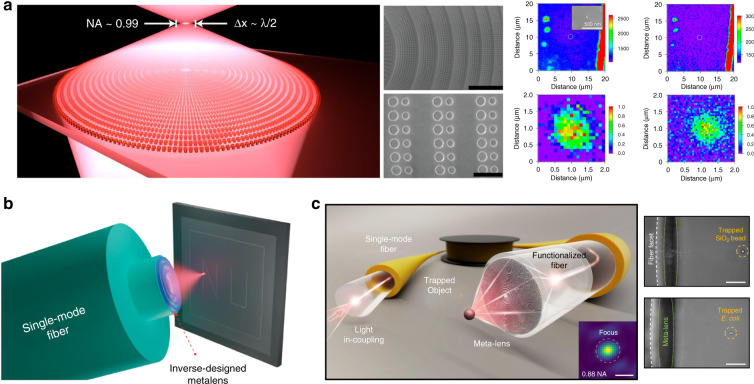
High-NA metalenses with methods relaxing the lattice constraints. **a** The schematic of the a-Si metalens (NA ~0.99) achieved by asymmetric scattering of dimer nanoantennas, and its application in a confocal map of photoluminescence. Reproduced with permission^49^. Copyright 2018, American Chemical Society. **b** The metalens (NA ~0.85) functionalized homemade two-photon direct laser lithography setup. Reproduced with permission^50^. Copyright 2021, American Chemical Society. **c** Metalens-based (NA ~0.88) fiber tip for optically trapping. Reproduced with permission^52^. Copyright 2021, the Authors, published by Springer Nature

The aforementioned lattice constraint could be broken with several methods. For example, Paniagua-Domínguez et al.^49^ designed a metalens based on diffracted energy redistribution employing amorphous silicon (a-Si) nanoantenna inclusions with asymmetric scattering patterns (Fig. 5a). The designed lens is applied in a confocal configuration to map color centers in sub-diffractive diamond nanocrystals. Inverse design methods are another candidate to design high-NA metalens since the freeform structures have no limitation of phase sampling (details in section “Advanced design methods”). Benefiting from the small dimension, an inverse-designed metalens is fabricated on a port of an optical fiber tip via 3D nanoprinting. The metalens functionalized fiber device is used for a homemade two-photon direct laser lithography setup^50^ (Fig. 5b). At the operating wavelength of 980 nm, the concentric circular structure offers a large NA (~0.85), a focal length (~8 μm), and a small written width (~200 nm). In addition to the discussion of metalens-incorporated optical tweezer^51^, the unique properties of the metalens-based fiber tip are demonstrated by optically trapping freely diffusing micro-objects in water (NA ~0.88)^52^ (Fig. 5c).

##### Limited focusing efficiency

The focusing efficiency is determined by the ratio of integral power at the focusing spot and the incident optical power. To achieve high focusing efficiency, high transmission and tight focusing of the transmitted wave are simultaneously required. The former is vital to avoid image defects such as flare spots, while the latter promises high contrast. Hence, lossless materials possessing high refractive indices within the bandwidth of interest are preferred. With a high refractive index, the electromagnetic field is strongly confined within the nanostructures, enhancing the phase-modulation. Singlet metalenses with focusing efficiency over 90%^34,53–55^ have been demonstrated. In particular, the NIL Technology company has reported silicon metalenses possessing 94% efficiency at 940 nm wavelength with anti-reflection coating on the non-structured side of the glass substrate. However, the NAs of these high-efficiency metalenses are smaller than 0.2.

The fundamental trade-off between the NA and efficiency can be interpreted by the following physical intuition. The diffracted angle of light at the edge of metalens increases with NA, requiring the increase in phase gradient. Then phase errors due to the coupling among adjacent elements are inevitable, and phase discretization level within the limited period should be reduced. Both factors lead to a decrease in diffracted efficiency at the edge, and the focusing efficiency thus decreases. Faced with the great demand for metalenses with high efficiency and large NA in state-of-the-art imaging devices, some design approaches are developed, including adjoint optimization^56^, grating averaging technique^57^, patching methods^58^, etc. However, there are still challenges such as large computational resources and boundary discontinuities. Hence, innovative methods for designing high-NA metalenses with high focusing efficiencies are in demand.

#### Wide-angle imaging

Wide-angle imaging is required in imaging applications such as AR, microscopy, landscape imaging, and image projection. Nevertheless, the FOV is often limited by the off-axis aberrations, in particular, the coma aberration. Aplanatic metalenses, which correct both spherical and coma aberrations simultaneously, are highly desired. As depicted in Fig. 4a–d, spherical wavefronts with different centers are required for free of axis and off-axis aberrations, respectively. Therefore, aplanatic imaging is challenging with conventional optics. To balance the axis and off-axis of aberrations, a bulky lens kit with cascading lenses is the conventional manipulation. Patterning meta-atoms on a curved surface have been demonstrated to reduce the volume^11^, but it poses inherited drawbacks of conventional lenses as well. For further miniaturization, flat metalenses to ideally focus light with arbitrary incident angle *θ*_i_ are desired, and the corresponding ideal phase profile should be a function of incident angle^59^:

5 $$\varphi \left( {r,\lambda ,\theta _i} \right) = - \frac{{2\pi }{n_b}}{\lambda }\left( {r\sin \theta _i + \sqrt {\left( {r - f\tan \theta _i} \right)^2\, +\, f^2} - \frac{f}{{\cos \theta _i}}} \right)$$

**Fig. 6:**
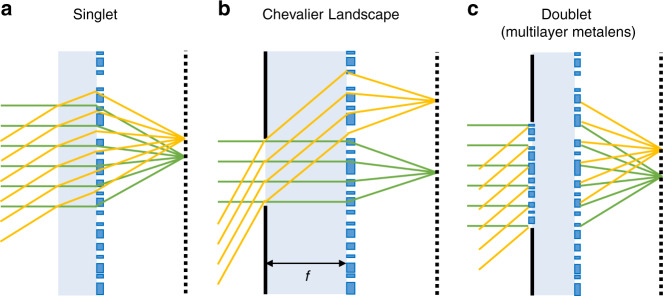
Three types of configurations for aplanatic imaging. **a** Singlet aplanatic metalens. **b** Chevalier Landscape metalens. **c** Doublet aplanatic metalens

Note that Eq. (5) yields a hyperboloidal phase profile [Eq. (1)] when *θ*_*i*_ = 0. However, the phase shift is usually angle-independent in common designs of metalenses. Alternatively, aplanatic metalenses with three main types of configurations are proposed to achieve wide-angle imaging (Fig. 6).

**Fig. 7:**
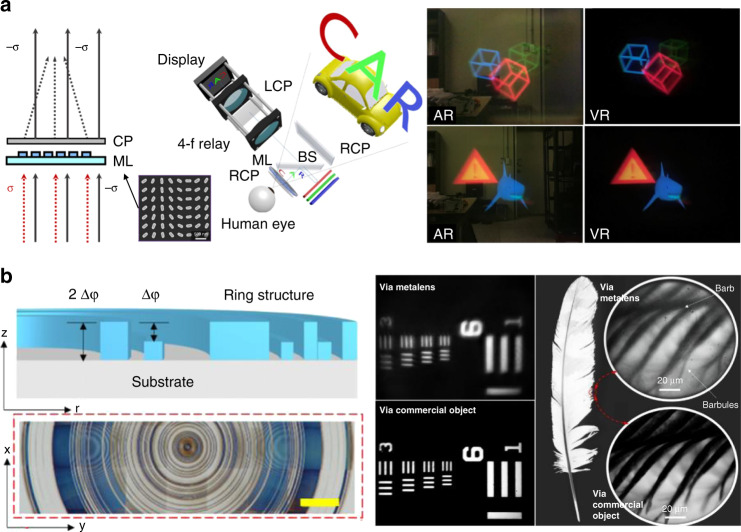
Representative singlet aplanatic metalenses and the corresponding applications. **a** The metalens eyepiece with FOV of 90° for full-color AR and VR display. Reproduced with permission^62^. Copyright 2018, the Authors, published by Springer Nature. **b** The single-layer aberration-compensated metalens with concentric nanoring structures for microscopy imaging. Reproduced with permission^64^. Copyright 2020, Wiley-VCH

The first type is the singlet metalens with hyperbolic^60–62^ or quadratic phase^63^ profiles (Fig. 6a). In this case, the FOV is limited by the increased WAF_rms_ with the incident angle, and further manipulation is required for wide-angle imaging. For instance, to achieve wide-field microscopy imaging with a FOV of 18°, a metalens array is developed to cover a wide area of CMOS image sensors so a full stitched wide-field image is completed^60^. Through a nanoimprinted large-area singlet metalens, Lee et al.^62^ experimentally demonstrated AR and VR near-eye displays (Fig. 7a). The metalens is constructed by spatially rotating poly-Si nanorods and shows full-color imaging with a wide FOV near 90° with the assistance of three dichroic mirrors. With circularly polarized incidence, the cross-polarized component providing a virtual image is focused while the co-polarized component carrying real-world scenes directly transmits^62^. Another kind of singlet aplanatic metalens is the concentric nanoring structures fabricated using the 3D printing technique by Qiu’s group^64^ (Fig. 7b). The vertical sizes and heights of the nanorings are designed with an epsilon-greedy algorithm-based scheme, achieving a full FOV of 32°. Through the microscopic images of the USAF-1951 target and a bird-feathers sample, the imaging quality of the fabricated metalens is comparable with the commercial plano-convex lens.

**Fig. 8:**
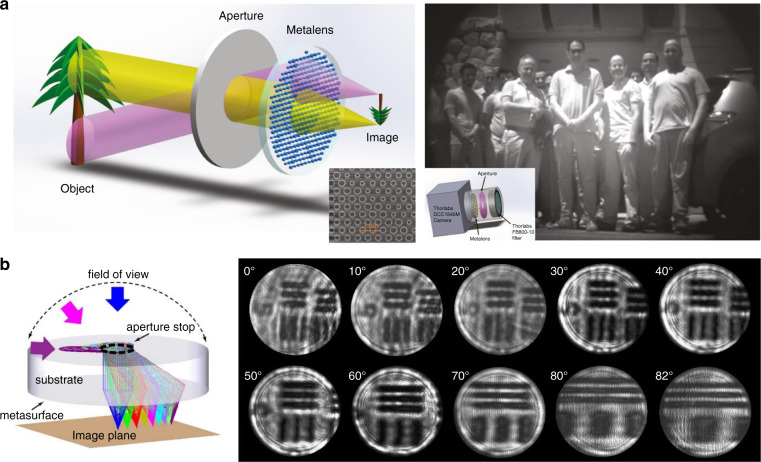
Chevalier Landscape metalenses. **a** Single-layer Huygens metalens for outdoor NIR imaging with FOV = 30°. Reproduced with permission^65^. Copyright 2020, the Authors, published by De Gruyter. **b** Fish-eye singlet metalens offering an ultra-wide FOV over 170°. Reproduced with permission^66^. Copyright 2020, American Chemical Society

The second type is the Chevalier Landscape metalens (Fig. 6b). Normally and obliquely incident rays are separated by the small aperture located at the front focal plane of metalens and then focused by different parts of the metalens, correcting all on- and off-axis aberrations simultaneously^65,66^. With the method, Uriel Levy’s group achieved a compact camera for wide-angle outdoor NIR imaging (FOV ~30°) with a Huygens metalens patterned by a-Si nanopillars^65^ (Fig. 8a). Quadratic phase function is used, and the sufficient focal length (3.36 mm) provides the higher angular resolution necessary for the recognition of facial features at a distance of several meters. Using a polynomial phase profile instead, Hu’s group demonstrated a fish-eye metalens operating at 5.2-μm wavelength^66^ (Fig. 8b). A Huygens metalens is constructed by rectangular and H-shaped PbTe blocks and fabricated on a 2-mm-thick CaF_2_ planar substrate. The other side of the substrate is a 1-mm-diameter circular aperture. The resolved images from 0° to 82° show the capability of the metalens to perform diffraction-limited imaging over an unprecedented FOV near 170°. Such a wide-angle single-piece metalens has no bulk counterpart, but the configuration would be still bulky with a large focal length.

**Fig. 9:**
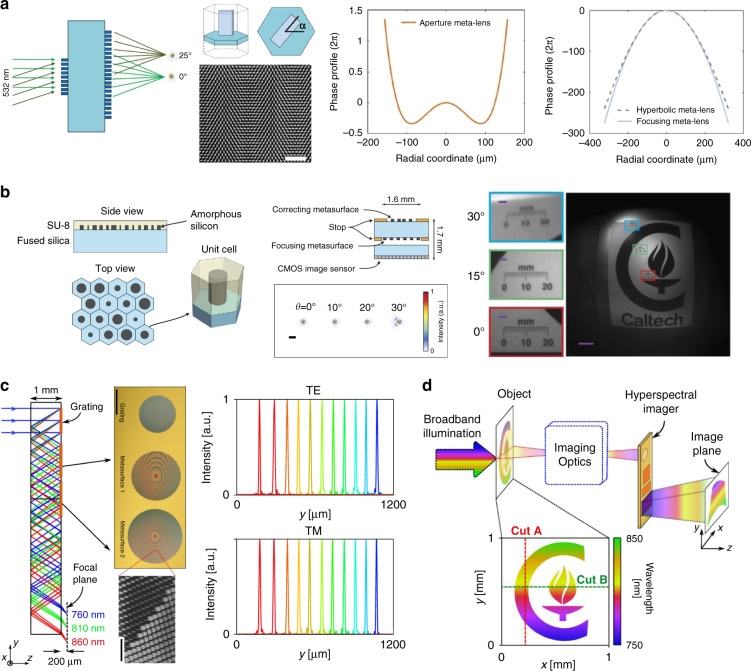
Aplanatic multilayer metalenses. **a** Doublet metalens based on PB phase offering 50° FOV with circularly polarized illumination and the phase profiles of two metasurfaces. Reproduced with permission^67^. Copyright 2017, American Chemical Society. **b** Doublet metalens based on dynamic phase offering 60° FOV with non-polarized illumination. Reproduced with permission^69^. Copyright 2016, The Authors, published by Springer Nature. **c** Compact spectrometer based on metalens-offering wide FOV near 68°: schematic of design and the ray-tracing result (left), the fabricated samples (middle), and the experimental results (right). Reproduced with permission^70^. Copyright 2018, The Authors, published by Springer Nature. **d** Hyperspectral imaging of a target with the folded metasurface hyperspectral imager and a tunable laser. Reproduced with permission^71^. Copyright 2019, American Chemical Society

The third type is multilayer metalens. In particular, the doublet configuration includes a corrector metasurface paired with a focusing phase profile (Fig. 6c). Capasso’s group utilizes TiO_2_ nanofins patterned on opposite silica substrate surfaces according to the PB phase (Fig. 9a). The doublet aplanatic metalens offers a NA of 0.44 and a 50° FOV with circularly polarized illumination at the working wavelength of 532 nm^67^. Achromatic aplanatic metalens has also been proposed combining this approach and the dispersion engineering method^68^. Similarly, Faraon’s group constructs a doublet metalens comprising of a-Si nanopillars cladded by a SU-8 polymer protection layer, which shows nearly diffraction-limited focusing for incident angle smaller than 30°. Then a conceptional miniaturized planar camera (total size: $1.6\;{{{\mathrm{mm}}}} \times 1.6\;{{{\mathrm{mm}}}} \times 1.7\;{{{\mathrm{mm}}}}$) is realized using the doublet metalens and a CMOS image sensor, offering a wide FOV imaging with unpolarized light^69^ (Fig. 9b). Working similar to the doublet metalens, two reflective metalenses are integrated side-by-side in a compact spectrometer in millimeter-scale volume^70^. The lights with different wavelengths are diffracted by the first grating (diffractive angle up to 33.9°) and then are collimated by the subsequent metalenses. As a result, the spectrometer has a resolution of about 1.2 nm, resolving more than 80 spectral points from 760 to 860 nm^70^ (Fig. 9c). Combining an additional transmissive metalens, a push-broom high spectral imager is further demonstrated^71^ (Fig. 9d). However, precise alignment is challenging in these aplanatic configurations.

#### Compromise between high-NA and high FOV

**Fig. 10:**
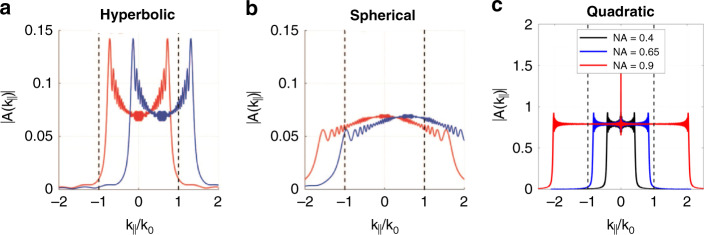
Fourier transforms (FT) amplitudes of the field generated by metalenses providing three different phase profiles. **a** FT spectra for a hyperbolic metalens. **b** FT spectra for a spherical metalens. **c** FT spectra for quadratic metalenses with different NA. In panels **a** and **b**, the red and blue lines correspond to normal and oblique incidence, respectively. Reproduced with permission^72^. Copyright 2019, Optical Society of America. Panel **c** is reproduced with permission^63^. Copyright 2020, American Chemical Society

High-NA metalenses usually utilize hyperbolic phase profiles for free-spherical-aberration, but the FOV is limited as coma is inevitable with oblique incidence, accompanying the decreased focusing efficiency^72^. The behind physics can be revealed with the Fourier transform (FT) spectrum of the field distribution immediately after the metalens^63,72^. Only *k*-vector components between –*k*_0_ and *k*_0_ are leaky and contribute to focusing, and the symmetry in this efficient space leads to the symmetry of the focal spot. As shown in Fig. 10a, the FT components for hyperbolic phase profiles are tightly confined inside the light line for normal incidence, and the dominant contribution of large *k* ensures a tight focal spot. Nevertheless, the coma is obvious when the large-*k* component is shifted beyond the effect space with an oblique angle. Because the amplitude of the large-*k* component increases with NA^72^, the achievable FOV decreases with increasing NA. Hence, an approach to enlarging the FOV of hyperbolic metalens is to reduce the NA, as adopted in some of the aforementioned multilayer and Chevalier Landscape metalenses. To maintain the high NA, the phase profile can be optimized by superimposing a term such as a polynomial function^73^ or be replaced by spherical or quadratic phase profiles^63^. They offer flatter FT spectra than the ideal hyperbolic counterparts, showing larger tolerance to the shift caused by oblique incidence (Fig. 10b, c). However, the on-axis resolution is compromised due to the broad spectra going beyond the effect space. Although the resolution can be increased with designs such as an aplanatic superoscillatory metalens, the focusing efficiency is limited by the sidelobe^74^.

Several advanced design methods such as adjoint optimization and catenary optics have been discussed for high-performance wide-angle metalenses^75^. In addition, exploiting the angular dispersion capability of metasurfaces would efficiently manipulate the on-axis and off-axis aberrations. By controlling the coupling between meta-atoms and the radiation properties of constituent meta-atoms, angle-dependent phase shift is achieved by meta-devices with metal-insulator-metal configuration^76,77^ and multilayered structures^78,79^. Sell et al.^80^ demonstrated a freeform silicon-based metagrating that can be continuously adjusted from linear to elliptical birefringence by changing the angle of incidence. Although the overall phase shift is not focused on in this work, it is angle-dependent according to the Jones matrix analysis.

### Chromatic aberration correction

Chromatic aberration occurs when light rays passing through a lens focus at frequency-dependent points due to optical dispersion. Chromatic aberration elimination at discrete wavelengths has been achieved through spatial multiplexing^34,81–83^. Multiple sets of meta-atoms are interleaved or stacked, with each controlling the wavefront of a wavelength. Meanwhile, broadband achromatic focusing is achievable by the dispersion engineering capability of metalenses.

#### Dispersion engineering for achromatic imaging

Metalenses introduce dispersion that is dictated mainly by the geometrical parameters and arrangement of the subwavelength structures^7,84^. As we know, the metalens exhibits different phase profiles depending on the wavelength of the incident light, and the Taylor expansion gives:

6 $${\upvarphi}\left( {r,\omega } \right) = {\upvarphi}\left( {r,\omega _d} \right) + \left. {\frac{{\partial {\upvarphi}\left( {r,\omega } \right)}}{{\partial \omega }}} \right|_{\omega _d}\left( {\omega - \omega _d} \right) + \left. {\frac{{\partial ^2{\upvarphi}\left( {r,\omega } \right)}}{{2\partial \omega ^2}}} \right|_{\omega _d}\left( {\omega - \omega _d} \right)^2 + \ldots$$

The first term on the right-hand side corresponds to the transmitted wavefront with the design frequency *ω*_*d*_, and it can be engineered by the phase control methods introduced in section “Phase modulation mechanisms”. The higher-order derivative terms including the second and the third terms ($\frac{{\partial {\upvarphi}\left( {r,\omega } \right)}}{{\partial \omega }}$ and $\frac{{\partial ^2{\upvarphi}\left( {r,\omega } \right)}}{{2\partial \omega ^2}}$ represent group delay and group delay dispersion profile, respectively) control the chromatic focal length shift of the metalens^85^. By simultaneously manipulating the phase, group delay, and group delay dispersion profiles, achromatic and chromatic applications are both demonstrated. On the one hand, the high diffractive dispersion is utilized for spectral tomographic imaging negating chromatic aberrations^86^, and a metalens-integrated nano-optic endoscope is presented for optical coherence tomography application^87^. On the other hand, achromatic imaging over broad bandwidth has been realized in the VIS^85,88,89^, NIR^55,90,91^, and MIR^92–96^ bands.

**Fig. 11:**
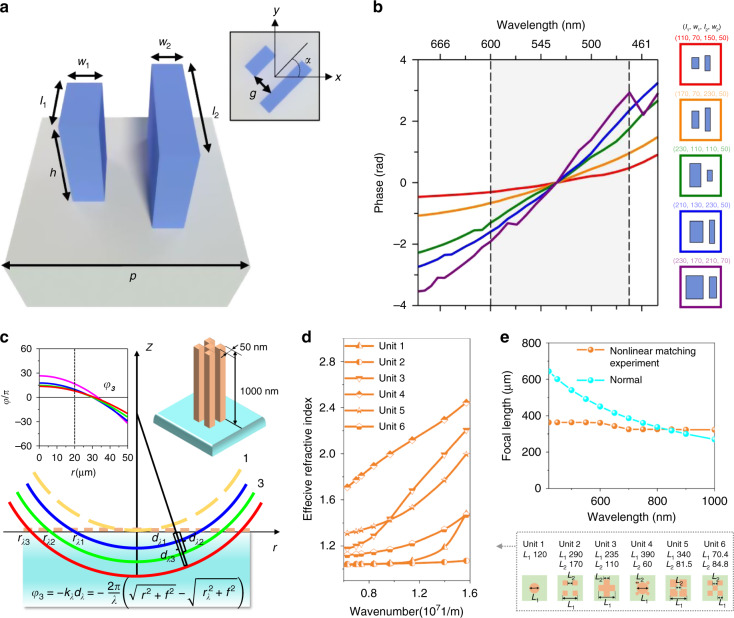
Dispersion engineering capability of meta-atoms. **a** Schematic of a metalens element for visible achromatic imaging. **b** Phase spectra for five different elements showing the tunability of the group delay by changing the lengths and widths of nanofins. Each colored curve corresponds to its element schematically shown on the right. **c** The schematic wavefronts of a nonlinear phase compensation scheme. **d** Effective refractive index spectra of six meta-units listed on the right. **e** Focal length distribution at different wavelengths. The orange line is the focal length of the nonlinear matched metalens, and the blue line is the normal negative dispersion reference curve. Panels **a** and **b** are reproduced with permission^88^. Copyright 2018, The Authors, published by Springer Nature. Panels **c**–**e** are reproduced with permission^98^. Copyright 2021, The Authors, preprinted by arXiv

According to Eq. (6), the group delay (*GD*) for a hyperbolic phase profile is:

7 $$GD\left( r \right) = \frac{{\partial {\upvarphi}\left( {r,\omega } \right)}}{{\partial \omega }} = \frac{n_b}{c}\left( {f - \sqrt {r^2 + f^2} } \right)$$

and the corresponding group delay dispersion $\frac{{\partial ^2{\upvarphi}\left( {r,\omega } \right)}}{{2\partial \omega ^2}}$ is zero. To achieve achromatic imaging, the GD is compensated by structural dispersion. There are four cases for achromatic metalenses in terms of methods: (i) The transmitted wavefront and the structural dispersion are simultaneously tailored by changing the geometric parameters of resonant meta-atoms. (ii) The transmitted wavefront and the structural dispersion are simultaneously tailored by changing the geometric parameters of waveguide-type meta-atoms. (iii) The transmitted wavefront and the structural dispersion are controlled by rotating orientation (PB phase) and geometric parameters of resonant meta-atoms, respectively. (iv) The transmitted wavefront and the structural dispersion are controlled by rotating orientation (PB phase) and geometric parameters of waveguide-type meta-atoms, respectively. Particularly, cases (ii) and (iv) are more common as waveguide-type meta-atoms usually offer larger achievable GD compensation than the resonant counterparts^97^ (see discussion in section “Challenges for broadband achromatic imaging”). A representative work is shown in Fig. 11a. Each meta-atom is comprised of two TiO_2_ nanofins in close proximity, acting as a coupled waveguide. Their geometric parameters are designed for linear structural dispersion in the 470–600 nm band (Fig. 11b), and the wavefront is tailored by PB phase^88^. Nevertheless, for a larger relative bandwidth (${{\Delta }}\omega /\omega _d$), the structural dispersion relationship shows a strong nonlinearity leading to a notable increase in the compensation error. To better match with the nonlinear structural dispersion, Hu et al.^98^ constructed the dispersion model with a wavelength-dependent position (Fig. 11c). Using the nonlinear dispersive phase compensation (Fig. 11d), they achieved ultra-broadband (400–1000 nm) achromatic focusing (Fig. 11e).

**Fig. 12:**
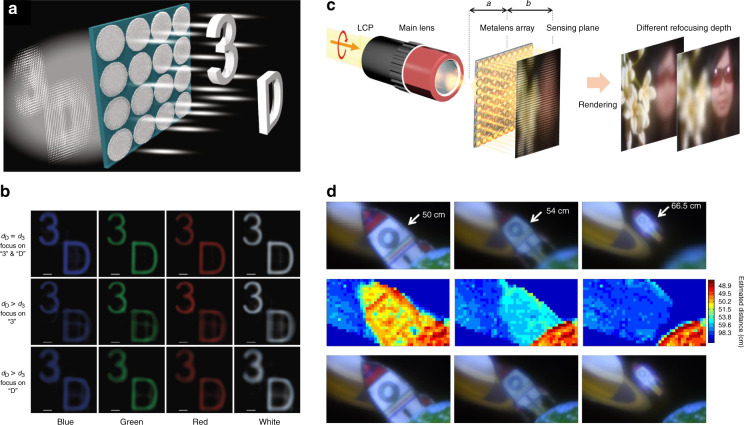
Applications of achromatic metalenses in lenslet-array-based imaging systems. **a** Schematic of integral imaging based on achromatic metalenses. **b** Reconstructed images in the cases of *d*_D_ = *d*_3_ (top row), *d*_D_ > *d*_3_ (middle row), and *d*_D_ > *d*_3_ (bottom row). **c** Schematic diagram of light-field imaging with metalens array and rendered images. **d** Rendered achromatic images focusing on the rocket with depths of 50, 54, and 66.5 cm (top row), the corresponding estimated depth maps (middle row), and rendered all-in-focus images (bottom row). Panels **a** and **b** are reproduced with permission^99^. Copyright 2019, The Authors, published by Springer Nature. Panels **c** and **d** are reproduced with permission^100^. Copyright 2019, The Authors, published by Springer Nature

The waveguide-type achromatic metalenses perform their potential in lenslet-array-based imaging systems. Achromatic projection of objects with different depths under white light illumination (430–780 nm) is achieved through an integral imaging system using SiN metalens array based on propagation phase^99^ (Fig. 12a, b). And an achromatic full-color light-field (LF) camera is demonstrated in the band 400–660 nm by a metalens array that captures LF information based on PB phase control of GaN nanoantennas^100^ (Fig. 12c, d).

#### Challenges for broadband achromatic imaging

Broadband achromatic focusing by a single metalens is an inspiring feature, but the achievable achromatic bandwidth is limited by some fundamental bounds and fabrication challenges.

##### Fundamental limit for ideal metalens

Regardless of the specific implementation, the achromatic bandwidth (${{\Delta }}\omega$) of a metalens is related to the time delay experienced by the signals (${{\Delta }}T$) and a dimensionless quantity $\kappa$ showing the upper bound of time-bandwidth products^101^:

8 $${{\Delta }}\omega \le \frac{\kappa }{{{{\Delta }}T}}$$

where the time delay ${{\Delta }}T$ compensates for the additional time taken by a signal arriving from the edge, and it is consistent with the group delay (i.e., ${{\Delta }}T(r) = GD\left( r \right)-GD\left( R \right)$) except in the presence of anomalous dispersion near resonances accompanied by strong absorption. Thus, the greatest delay is required at the center of the metalens and can be obtained according to Eq. (7). Then the upper bound on the achromatic bandwidth is determined as $\Delta \omega _{{{{\mathrm{max}}}}} = \kappa /{{\Delta }}T(0)$, and the fundamental limit of achromatic bandwidth is derived as^101^:

9 $$\Delta \omega \le \Delta \omega _{{{{\mathrm{max}}}}} = \frac{{\kappa c}}{{{n_b}\left( {\sqrt {f^2 + R^2} - f} \right)}} = \frac{{\kappa c\sqrt {1 - \left( {NA/n_b} \right)^2} }}{{n_b{f}\left[ {1 - \sqrt {1 - \left( {NA/n_b} \right)^2} } \right]}}$$

where $n_b$ represents the background refractive index. With increasing physical parameters of the metalens such as radius (*R*), focal length (*f*), and NA, a greater time delay is required, leading to the shrinking achievable achromatic bandwidth ($\Delta \omega _{{{{\mathrm{max}}}}}$). For metalens with specific *R* and *f*, the approach to increase achievable achromatic bandwidth is to enlarge $\kappa$, which is dependent on the type of the metalens.

For resonant-type metalenses ($H \ll \lambda$), the coupled-mode theory provides a geometry- and material-independent value ($\kappa = 2$) for a single resonant cavity^102^. Hence, the achromatic bandwidth of such metalenses is limited. The value may be enlarged with meta-atoms that contain multiple resonant cavities. Actually, multi-resonances have been found to support broadband achromatic reflective response^84,97,103,104^, but the efficiency is limited by the lossy metallic components. Multi-resonances in dielectric nanostructure should be focused on, and the behind physics merits investigation. Moreover, one needs to trade off the achromatic bandwidth against the transmission according to the impedance-matching limit, known as the Bode–Fano limit^101,105^: $\Delta \omega _{{{{\mathrm{max}}}}} = {-2\pi} {c}/\left[ {{{{\mathrm{log}}}}\left| \Gamma \right| H\left( {n_{{\rm{eff}}}^2 - n_b^2} \right)} \right]$, where *n*_eff_ is the effective refractive index, and *Г* is the in-band reflection coefficient.

For non-resonant dielectric meta-atoms acting as truncated waveguides ($H\sim \lambda$), Tucker et al.^106^ gave the upper bound value as $\kappa = {\upomega}_{{{\mathrm{d}}}}H/{{{\mathrm{c}}}}(n_{{{{\mathrm{max}}}}} - n_{{{{\mathrm{min}}}}})$, where $n_{{{{\mathrm{max}}}}}$ and $n_{{{{\mathrm{min}}}}}$ are the maximum and minimum effective indices. Miller et al.^107^ provided a limit that is valid for more generic cases (not necessarily dielectric) or those with larger thicknesses ($H \,> \,\lambda$): ${\upkappa} = \frac{1}{{2\sqrt 3 }}\omega _{{{\mathrm{d}}}}H/{{{\mathrm{c}}}}\left| {(n_{{\rm{max}}}^2 - n_{{{\mathrm{b}}}}^2)/n_{{{\mathrm{b}}}}^2} \right|$. In contrast to resonant-type metalenses, the non-resonant dielectric metalenses can offer larger fundamentally achievable achromatic bandwidth as the time delay is compensated for by structural dispersion of the effective waveguides. As discussed in Shrestha’s work^90^, since the waveguide-mode dispersion converges to the light line of the high (low)-index material at high (low) frequency, the dispersion is approximately

10 $${{\Delta }}\phi = \frac{H}{c}\left[ {n_{{\rm{eff}}}\left( {\omega _{{\rm{max}}}} \right)\omega _{{\rm{max}}} - n_{{\rm{eff}}}\left( {\omega _{{\rm{min}}}} \right)\omega _{{\rm{min}}}} \right]$$

Therefore, a large structural dispersion can be obtained with high refractive-index contrast (${{\Delta }}n = n_{{\rm{eff}}}\left( {\omega _{{\rm{max}}}} \right) - n_{{\rm{eff}}}\left( {\omega _{{\rm{min}}}} \right)$) and large height (*H*), automatically reaching the Turker’s limits.

##### Typical challenges for waveguide-type achromatic metalenses

Nevertheless, achieving the fundamental achromatic bandwidth is challenging from a fabrication perspective. Considering longer meta-atoms is an effective way to improve the bandwidth performance with transparent materials. However, if the height of a meta-atom is limited beyond a wavelength, its lateral size must be very small to form the effectively homogeneous slab, leading to a high ratio aspect, whose fabrication is still challenging. Another approach to enlarge the achromatic bandwidth is increasing the refractive-index contrast ${{\Delta }}n$. However, the decade-improvement in achromatic bandwidth is unlikely, even considering freeform all-area optimization, as the refractive-index contrast would not be over a magnitude due to the limited refractive indices of the transparent dielectric. Although large effective indices are enabled by hyperbolic photonic crystals^108^, heights comparable to several wavelengths are required, leading to high ratio aspects as well.

**Fig. 13:**
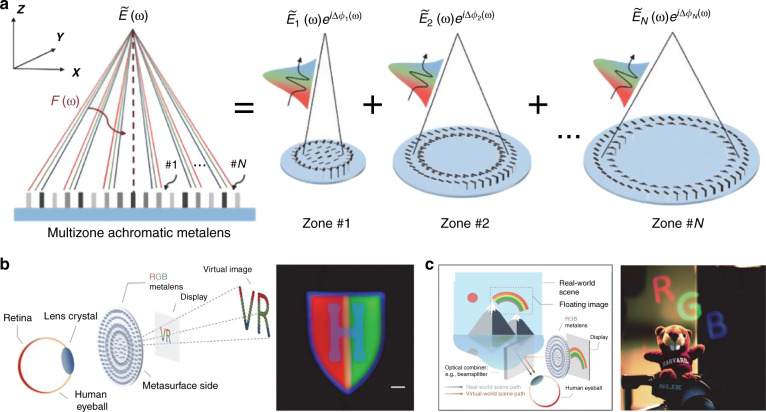
A 2-mm-diameter RGB-achromatic metalens for AR/VR applications. **a** Schematic drawing of the multizone RGB-achromatic metalens. **b** Schematic illustration of the VR mode (left) and the VR image (right). **c** Schematic illustration of the AR mode (left) and the AR image (right). Reproduced with permission^89^. Copyright 2021, The Authors, some rights reserved; exclusive licensee American Association for the Advancement of Science

Another restriction related to the fabrication challenge is the diameter size. According to Eq. (7), the required group delay at the metalens center increases with the diameter (2*R*). For instance, to achieve the group delay required by a 1-mm-diameter TiO_2_ metalens with NA = 0.1 for the VIS band, the height of the meta-atoms must be about 7.5 μm, implying tens or hundreds of aspect ratio. Therefore, discrete-wavelength-achromatic imaging based on spatial multiplexing^34,81–83^ or dispersive phase compensation^109^ is exploited instead for the large-diameter case. However, large computation resources are required in the multiple-objects design process. An approach to strike a compromise is to exploit constructive interference of light from multiple zones and dispersion engineering^89^ (Fig. 13a). With the method, a 2-mm-diameter RGB-achromatic metalens is practically applied in a VR/AR eyepiece (Fig. 13b, c). The achromatic imaging capability of the metalens makes the eyepiece more simple and lightweight compared with the previous work shown in Fig. 7a.

##### Accesses to relax the constraints

For a non-ideal metalens that does not achieve aberration-free focusing, the achromatic bandwidth may surpass the upper bounds ($\Delta \omega _{{{{\mathrm{max}}}}}$) since an error in the implemented phase profile would be acceptable^101^. Hence, a compromise should be manipulated between imaging quality and achromatic bandwidth.

In addition to considering multiple-resonances, multilayer metalenses^110–113^ that break the assumption of one-dimensionality may surpass the aforementioned bounds to some degree while remaining small aberrations. For instance, A hybrid achromatic metalens designed by combining recursive ray-tracing and phase libraries shows average focusing efficiencies greater than 60% over a broad NIR band (1–1.8 μm), while the refractive index of composite material (GaN) is about 1.5^110^. Furthermore, one can seek other approaches to realize achromatic focusing. For example, superoscillation, which is usually utilized for subwavelength super-resolution focusing, has also been reported as a holographic approach for achromatic focusing^114^. A binary amplitude mask is constructed by two sets of PB meta-atom with different sizes but sharing the same rotating regulation. The focused double-hump-shape intensity distributions along the propagation axis of different wavelengths overlap even though the focal centers are not in the same plane. Therefore, achromatic imaging could be realized in the overlapped area. However, the extension of the intensity distribution also leads to low efficiency. Reverse design methods and artificial intelligent algorithms would give further access to high-performance achromatic metalenses for practical applications.

### Multifunctionality

For some imaging applications such as polarization imaging, zoom imaging, and 3D imaging, simultaneous or tunable multi-foci are required. Cascading optical elements with different functionalities and sometimes mechanical components are thus required to work with lenses. Therefore, the difficulty to reduce the volumes of these imaging systems increases with the number of these separating components. The alignment as well the adjustment and matching of elements’ performances are also increasingly challenging with the element number. Although fewer elements are used in some optical systems using time-division strategies (for example, by rotating a polarizer for polarization detection), real-time measurements are unachievable. In contrast, multiple functionalities can be implemented within the same shared aperture of a single metalens due to the high flexibility in controlling light^115,116^. Hence, the multifunctionality of metalenses enables compact, simple, and real-time (if required) imaging systems by reducing both the element size and number. To illustrate the various opportunities of single-piece multifunctional metalenses to replace complicated systems, here we highlight some applications.

#### Real-time polarization imaging

The real-time polarization imaging technique, which simultaneously forms multiple polarization-resolved images, can reveal information that is invisible in traditional imaging by a single shot. Conventional optical setup for real-time polarization imaging involves a division of focal plane systems with multiple components, including beam splitters, polarizers, waveplates, cascading lenses, and multiple detectors^117^. Although the simplified systems have been demonstrated by incorporating a layer of polarizers above the photodiodes^118^ or paring a metagrating with a lens^119^, they suffer from low efficiency and limited image contrast, respectively. The polarization-assisted multifunctional metalenses offer a new platform for high-efficiency and compact polarization cameras.

**Fig. 14:**
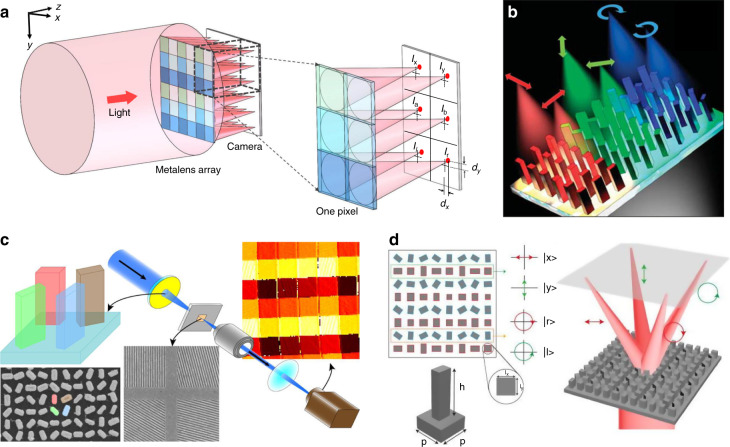
Single-piece multifunctional metalenses for real-time polarization imaging. **a** Scheme of the generalized Hartmann–Shack beam profiler. Reproduced with permission^124^. Copyright 2018, The Authors, published by Springer Nature. **b** Full-stokes imaging polarimetry based on a meta-unit with three parts (colors represent the polarization states). Reproduced with permission^125^. Copyright 2018, American Chemical Society. **c** The metalens-based illumination system of a polarization microscope. Reproduced with permission^126^. Copyright 2020, American Chemical Society. **d** The polarimetry for mid-infrared wavelength. Reproduced with permission^127^. Copyright 2020, AIP Publishing

Incident polarization-dependent transverse separation of focal spots is required for real-time polarization imaging. Birefringent effects in anisotropic meta-atoms are utilized for linear polarized (LP) waves^38,120,121^, and the geometric phase is widely used for circularly polarized (CP) waves^94,122,123^. Because the incident LP and CP waves cannot be simultaneously separated by the same meta-atoms due to structural symmetry, the full-polarization/full-stokes metalenses, which involve six foci, are mostly realized by combining several sets of sub-units. Yang et al. proposed a generalized Hartmann–Shack wavefront sensor by integrating a standard camera with a metasurface array whose superpixel contains six metalenses, each of which attributes the focus of one particular polarization state (Fig. 14a)^124^. In addition to the real-time full-polarization imaging, the sensor can simultaneously detect phase profiles by further analyzing the foci displacements. Faraon’s group divided the meta-unit into three parts, with each splitting an orthogonal polarization basis set^125^ (Fig. 14b). Consequently, the power in the six polarization states is measured by the corresponding image sensor pixel, offering a full-stokes measurement. Furthermore, the illumination system of a polarization microscope is demonstrated by a metalens whose unit is comprised of four nanofins on the SiO_2_ substrate, and each nanofin acts as a half-wave plate to control a linear polarization state^126^ (Fig. 14c). Similarly, Luo’s group achieved the simultaneous imaging of four polarization states at the MIR wavelength of 10.6 μm (Fig. 14d). Two sets of silicon pillars are interleaved. One forms convergent wavefronts of horizontal/vertical polarization by dynamic phase, while the other offers the CP-dependent transverse shift of focusing by combining the dynamic and geometric phase (i.e., the side length and the rotation angle of the silicon pillars are simultaneously adjusted)^127^.

#### Zoom imaging

Zoom imaging is usually performed by longitudinal motion of multiple lenses in conventional optics. The complex and bulky tunable lens kit, however, has been transformed into a single element with dynamically tunable metalenses. The methods for zoom metalens can be divided into the following three strategies.

**Fig. 15:**
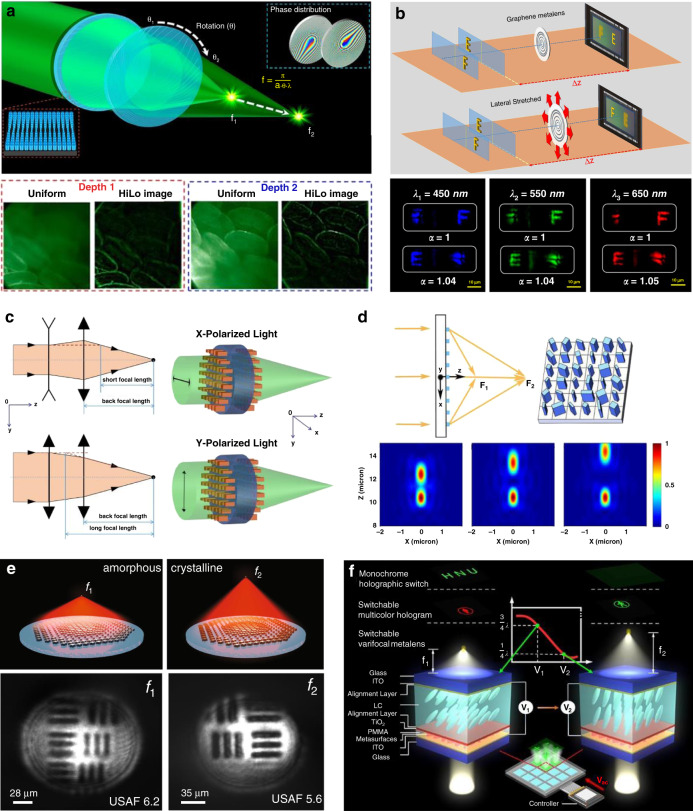
Strategies for zoom metalenses. **a** Variable focal length via microelectromechanical systems. Reproduced with permission^130^. Copyright 2021, American Chemical Society. **b** Continuous tuning of the focal length by stretching elastomeric materials. Reproduced with permission^135^. Copyright 2018, The Authors, some rights reserved; exclusive licensee American Association for the Advancement of Science. **c** Doublet metalens offering dual-step zoom switched by the linear polarization state of the incident light. Reproduced with permission^136^. Copyright 2019, Optical Society of America. **d** Singlet metalens with dual focal lengths switched by the circular polarization state of the incident light. Reproduced with permission^138^. Copyright 2019, Optical Society of America. **e** Dual-step zoom based on phase transformation of optical change material. Reproduced with permission^146^. Copyright 2019, The Authors, published by Springer Nature. **f** Varifocal metalens based on tunable birefringent liquid crystals. Reproduced with permission^148^. Copyright 2021, American Chemical Society

(i) Zoom via mechanical deformation or displacement, which incorporates microelectromechanical systems^128–133^ or elastomeric materials^9,53,134,135^. For instance, the focal length of a dielectric Moiré metalens at 532 nm is changed from ∼10 to ∼125 mm by tuning the mutual angle between the two complementary phase plates composed of GaN meta-atoms^130^ (Fig. 15a). The designed telecentric configuration enables high-contrast multiplane fluorescence imaging, and optically sectioned images of ex vivo mice intestine tissue samples are demonstrated. As shown in Fig. 15b, focal length tuning for a multi-color channel is demonstrated by laterally stretching a varifocal graphene metalens. An over 20% focal length tuning range is achieved for red (650 nm), green (550 nm), and blue (450 nm) light, and zoom imaging of different objects located along the axial direction is demonstrated^135^. Furthermore, with tunable metalens controlled by electrically engineered artificial muscles, monochromatic aberration corrections are simultaneously performed while continuously adjusting the focal length^53^.

(ii) Step zoom via polarization tuning, where the focal length is dependent on the polarization states of the incident light^136–142^. Dual-step zoom is achievable with the LP-dependent or CP-dependent response of multilayer metalenses^136,137^. Fu et al. proposed a double-sided metasurface utilizing dynamic phase modulation of anisotropic silicon nanobricks^136^. For the operating wavelength of 658 nm, the effective focal length switches by the LP of the incident beam, as the front-metasurface offers convergent and divergent functions for the orthotropic LPs, respectively (Fig. 15c). However, such a multilayer metalens suffers relatively low focusing efficiencies (<20%) and more difficult fabrication in comparison to singlet metalenses offering multi-step zoom function^138,139,143^. Continuous focal length tuning by changing the LP state of incidence is also achieved^140^, with the tuning rang smaller than the depth of focus of the metalens. Combining dynamic and geometric phases, Tian et al. proposed a metalens offering two CP-dependent foci with high efficiency (up to 72%), whose relative intensities can be modulated conveniently by changing the ellipticity of the incident light^138^ (Fig. 15d).

(iii) Zoom via reconfigurable material, whose optical properties can be changed by non-mechanical actuation methods such as optical, thermal, and electrical tuning. So far, multi-foci metalenses have been realized by using optical phase change materials (OPCM)^144–146^, liquid crystals (LC)^147–151^, nonlinear materials^152^, thermo-optical materials^153,154^, reconfigurable polymers^155^, etc. Particularly, OPCMs offer a promising route for realizing zoom metalens owing to the extremely large refractive index contrast associated with material phase transformation^156–160^. Yin et al.^145^ patterned two groups of the plasmonic antenna on top of a blanket OPCM film, with each group responding to phase control of incident light at either the amorphous or crystalline state of the film. However, focusing efficiencies are limited (5% and 10% for the two states, respectively) by the shared-aperture layout and the use of metallic meta-atoms. Shalaginov et al.^146^ demonstrated an OPCM-based all-dielectric Huygens metalens that is optimized via a generic design methodology. The focal length can be switched between 1.5 mm (at amorphous state) and 2 mm (at crystalline state) by electrical tuning, with the focal efficiency over 20% at the operating 5.2 μm wavelength. Using the bi-foci metalens as an objective in a multi-depth imaging system, well-resolved images of standard USAF-1951 resolution charts coinciding with the two foci are both captured (Fig. 15e). LC-based metalenses are also presented due to the maturity of LC materials^147,148^. For instance, Hu et al.^148^ demonstrated electrically tunable multi-zoom metalens by integrating birefringent nematic LCs with a PB phase-based metasurface (Fig. 15f). By applying different voltages, the orientation of LC molecules is adjusted to realize a variable wave plate with different phase retardations.

#### 3D imaging

LF imaging is an approach for single-shot 3D imaging without entailing any physical moving parts. In an LF imaging camera, a lenslet array is employed to capture the images from different vantage points. The depth information of the object is captured as the pixellated image and can be reconstructed slice by slice from a series of rendered images with different depths of focus. The monochromatic and achromatic aberrations in LF imaging can be corrected by specific algorithms^161^ and dispersion engineering capability of metalenses^100^, respectively. However, there is still an inherent trade-off between spatial and angular resolution in conventional LF imaging. As the spatial density of the lenslet-array increases, the spatial resolution increases, while the angular resolution is limited by the reduced aperture of each lens.

**Fig. 16:**
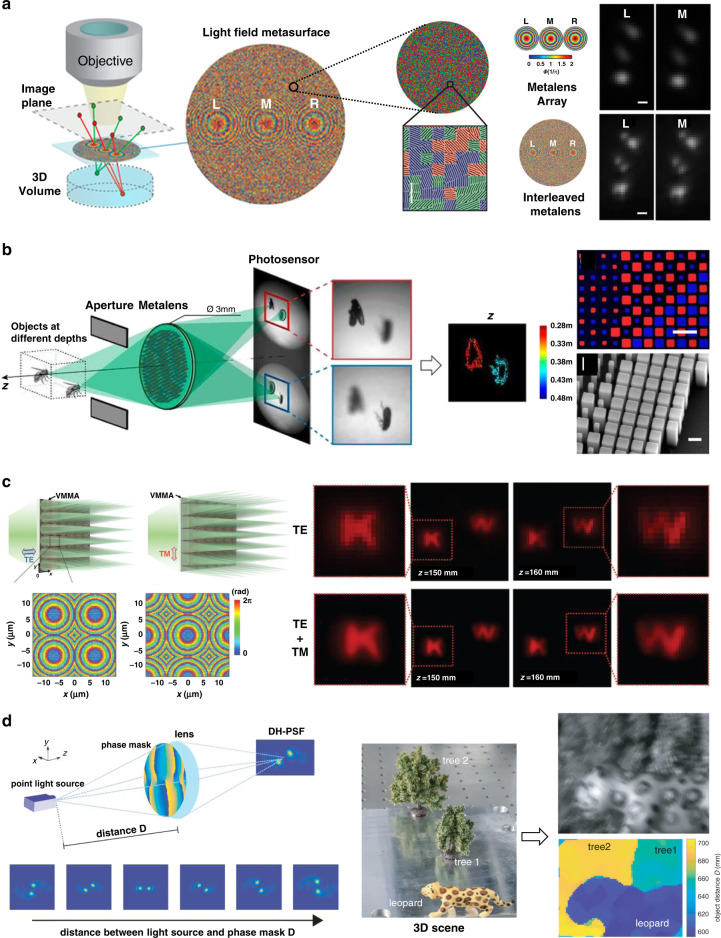
Metalenses for single-shot 3D imaging. **a** Layout scheme of the spatially interleaved metalenses with the zoomed-out false-colored SEM (left), and the comparison of imaging results (right). Reproduced with permission^162^. Copyright 2019, American Chemical Society. **b** A depth sensor based on a metalens simultaneously capturing two images with different defocus, insets are enlarged view of false-colored design (top) and side-view SEM image (bottom). In the right figure, meta-atoms with different colors correspond to distinct focusing phase profiles of the interleaved lenses. Reproduced with permission^163^. Copyright 2019, National Academy of Sciences. **c** Schematic configuration of the VMMA for TE/TM beam and the corresponding transmission phase profile (left); reconstructed depth-slice images for incidence with one and two polarizations (right). Reproduced with permission^164^. Copyright 2020, Wiley-VCH. **d** Schematic of 3D imaging with a double-helix-PSF rotating with the object distances (left) and the experimental result: photo of a 3D scene (middle), raw image (top in the right), and the retrieved depth information (bottom in the right). Reproduced with permission^165^. Copyright 2019, The Authors. Published by SPIE

An approach to mitigate this issue is to interleave the metalenses. As a proof of concept, Holsteen et al.^162^ proposed a metalens containing three interleaved phase profiles. The three sets of meta-atoms are arranged according to the PB phase and are randomly interleaved within a 200-μm-diameter shared aperture. As depicted in Fig. 16a, the depth information on particles is directly translated to lateral information in the sub-images, and the resolution is doubled compared with the same non-interleaved three metalenses. Synchronous 3D imaging without substantially modifying the optical system of a conventional optical microscope is further demonstrated simply by adding a patterned coverslip to the top of a fluorescent sample specimen. Another example is the passive snapshot depth sensor demonstrated by Capasso’s group^163^. The 3-mm-diameter metalens is comprised of two sets of meta-atoms. By applying cutting-edge nanotechnology and computer vision algorithms to two differently defocused images, depth measurement over a 10-cm distance range is achieved (Fig. 16b).

Park et al.^164^ focused on enhancing the spatial resolution of LF images by a virtual moving metalens array. By capitalizing on resonant a-Si:H meta-atoms that impart polarization-dependent phase, the sampling position is laterally shifted by half the elemental lens pitch by switching the linear polarization state of incidence (Fig. 16c). Compared with the normal case (demonstrated by only TE illumination), combining two sets of images (with TE and TM illumination, respectively) enhances the image resolution with the same aperture, so the spatial resolution of the reconstructed depth-slice images is increased with no angular resolution degradation.

3D imaging is also demonstrated via a single-aperture lens implemented by a polarization-insensitive Huygens’ metasurface phase mask^165,166^. As the generated double-helix point spread function (PSF) rotates with the object distances, the depth information of the 3D scene is retrieved with the captured raw image^165^ (Fig. 16d).

#### Limitations of multifunctional metalenses

In addition to the applications that have been introduced in this review, there are multifunctional metalenses for nanotracking^167^, hologram^168^, optical analog computing^169^, and phase measurement^170,171^ applications. However, multifunctional metalenses usually involve multiple focal spots, so several sets of sub-unit are usually interleaved to achieve the multiple functions within a shared aperture. Hence, the overall efficiency is limited by not only the efficient component of power but also the crosstalk among the sub-units. Balancing the performances among different focal spots is also a tough task. To optimize the multifunctional metalens, a new criterion could be proposed for the overall evaluation. For example, the performance of the ratio of average focal efficiency ($\bar \eta$) to the root-mean-square deviation of the focal efficiencies ($\eta _{{{{\mathrm{rms}}}}}$) of the multiple focal spots, i.e., ${{{\mathrm{FOM}}}} = \bar \eta /\eta _{{{{\mathrm{rms}}}}}$. To speed up the optimization, intelligent algorithms would be the right hand^172–174^.

## Common challenges for further development

In addition to the challenges concerning typical performances, there are some common challenges for the metalens-integrated applications as the following discussion.

### High-efficiency design methods

#### Limitations of the conventional design approach

The high degree of freedom enables versatile functionalities and high performances of metalenses. Nevertheless, extremely high memory space is required in the conventional design approach of metalenses. As introduced in step (ii) in the section “Principle”, the nanostructure-phase library is constructed by exhaustively scanning all parameters of meta-atoms (including the geometry, size, constituent, etc.) and incident beam (including polarization, incident angle, etc.). Therefore, the required simulation time explosively increases with the number of design degrees of freedom.

Moreover, there are conflicts in different performances of metalenses as mentioned above: The high NA and large FOV are incompatible due to the trade-off between on-axis and off-axis resolution; the breakthrough broadband achromatic feature of a single metalens suffers from the compromises among the operating bandwidth, the focusing efficiency, diameter, and the structural complexity; the further applications of multifunctional metalenses involve the optimization for multiple objectives. These problems lead to multi-objective tasks that further extend the memory space requirement.

Another challenge is the overall optimization of metalens-based imaging systems. The conventional key indicators for lens systems such as PSF, SR, modulation transfer function, and signal-noise ratio are also suitable to characterize metalenses. These criteria can be directly measured^65^ or calculated with the measured phase profile^171^. The criteria could efficiently help the optimization of metalenses in optical systems, which could be performed with some commercial software such as Zemax involving ray tracing and wave optics. Note that the design of metalens typically relies on numerical programs solving full-wave Maxwell equations. Zemax-interoperability has been enabled by Lumerical, with the nearfield data directly imported into the ray-tracing solver. VirtualLab Fusion provides another approach to designing metasurfaces by combining fast physical optics and ray/field-tracing solvers. In the overall optimization, however, efficient loop data transfer and iteration are arduously achieved without the assistance of customized codes for the moment.

#### Advanced design methods

##### Intelligent design

The simulation requirement in computation resources can be reduced with the assistance of machine learning algorithms such as neural networks^175–178^. For instance, a phase library containing 15,753 meta-atoms is generated in less than one second by a backpropagation neural network^179^. However, the massive dataset is expensive and sometimes unrealistic regarding the economic and labor costs. To release the computation pressure, artificial intelligence methods requiring smaller training datasets (such as transfer learning^177^ and reinforcement learning^180^ methods) could be particularly considered.

**Fig. 17:**
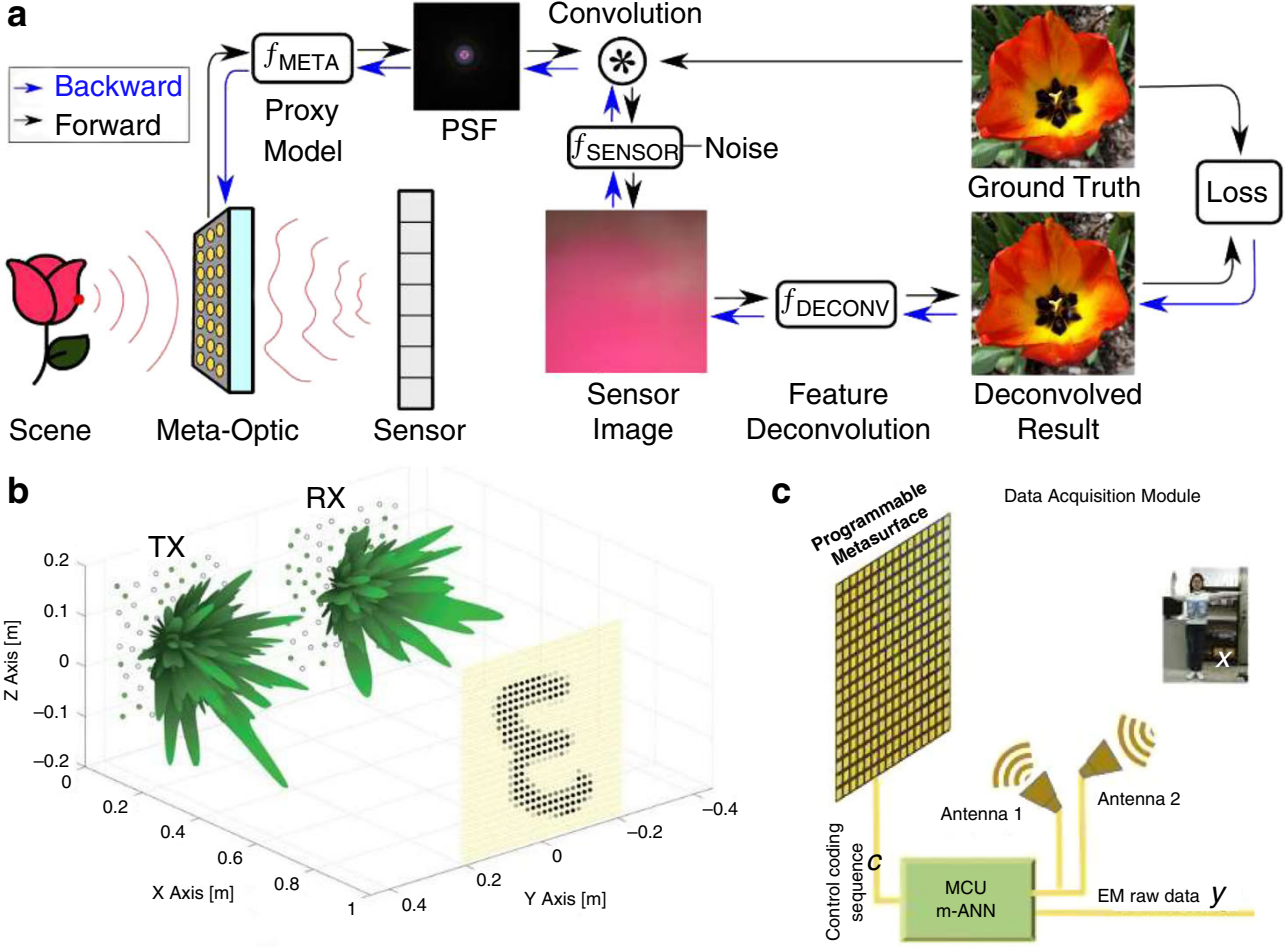
Intelligent end-to-end method for metalens global design. **a** The proposed end-to-end imaging pipeline for the nano-optics imager via neural computational imaging. Adapted with permission^181^. Copyright 2021, The Authors, published by Springer Nature. **b** Sensing setup with a metasurface (TX) illuminating the metallic digit in free space and a second metasurface (RX) capturing the reflected waves. Adapted with permission^184^. Copyright 2019, The Authors, published by Wiley-VCH. **c** Setup for the data acquisition module of the intelligent sensing system. Adapted with permission^185^. Copyright 2020, The Authors, published by Elsevier

The intelligent methods are also helpful in overall design and optimization. Tsenga et al.^181^ presented a high-quality, nano-optics imager via neural computational imaging. They devised a fully differentiable learning framework that learns about a metasurface physical structure in conjunction with a neural feature-based image reconstruction algorithm. From the optimizable phase profile, the differentiable model produces spatially varying PSFs, which are then patch-wise convolved with the input image to form the sensor measurement (Fig. 17a). The final image is produced by deconvolved sensor reading using a neural feature-based image reconstruction algorithm. After optimization, a metalens with a FOV of 40° and an f-number of 2 is experimentally demonstrated, providing full-color imaging on par with a bulky commercial lens. By combining reconfigurable meta-atoms, we may envision extending such neural nano-optics towards adaptively programable imaging and sensing devices. There have been intelligent imagers based on programmable metasurfaces through similar end-to-end pipelines^182^. The responses of programable meta-atoms contribute to the physical weights and they are jointly trained with the digital weights^183^. Using dynamic metasurface apertures capable of transceiving programmable microwave patterns, Hougne et al. numerically demonstrated a prototypical object recognition task^184^ (Fig. 17b). Subsequently, Li et al. reported the first experimental implementation of learned sensing, which completes a human gesture recognition task with a programmable metasurface reflect-array^185^ (Fig. 17c). However, both meta-imagers are realized with microwaves, whose corresponding sizes of meta-atoms are large enough for locally tunable response-controlling. Although the application at optical frequencies is still challenging, these intelligent electromagnetic meta-imagers may indicate important directions for dielectric metalens-based imaging systems.

##### Freeform all-area optimization

**Fig. 18:**
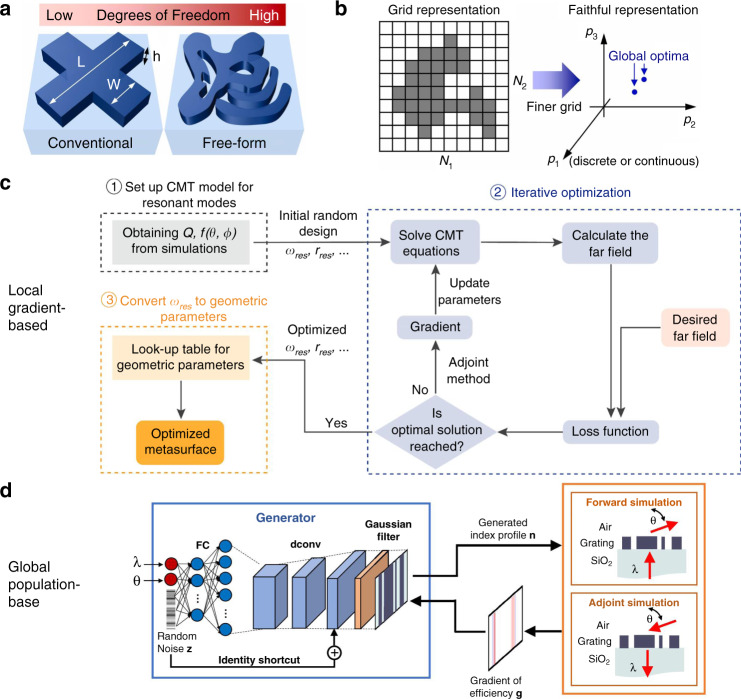
Freeform all-area optimization methods and the design flows. **a** Comparison between conventional (left) and freeform (right) design schemes. Reproduced with permission^189^. Copyright 2022, The Authors, published by De Gruyter. **b** A grid representation (left) approaching a faithful representation that coincides with the whole device design space (right). In a faithful representation (middle). Reproduced with permission^189^. Copyright 2022, The Authors, published by De Gruyter. **c** Metasurface design procedure using couple-mode-theory (CMT) and adjoint optimization. Reproduced with permission^191^. Copyright 2020, The Authors, published by Springer Nature. **d** Schematic of a global population-based optimizer for metagrating optimization. Reproduced with permission^193^. Copyright 2019, American Chemical Society

Freeform all-area optimization algorithms such as the topology optimization (TO) method^186–188^ are the alternative design method to avoid the aforementioned memory issue. Instead of fixed primitive shapes in conventional metalens designs, the meta-atoms have nontrivial shapes that can be topology-modified^189^ (Fig. 18a). TO method considers the dielectric permittivity at every spatial point as a design variable. The target is a faithful representation that coincides with the whole device design space, and a classical explicit representation is grid representation that approaches the target with finer grids (Fig. 18b). There are also implicit design representation methods that try to capture the “axes” of the true design space via domain expertise^189^.

In terms of the optimizers, local gradient-based and global population-based optimizers are included^190^. The local gradient-based optimizers leverage the adjoint variables method. An example is shown in Fig. 18c^191^. A physical model, the couple-mode-theory (CMT) model, is first built by previous simulations. The researchers then initialized the iterative optimization process with a randomly selected design and calculated the far-field. Using the adjoint method, the gradient of the loss function is calculated to update the design parameters. After the optimization, the corresponding geometric parameters of the resonators can be directly obtained by using the look-up table. In this method, a large-scale metalens (*R* ~10,000*λ*) with NA = 0.9 is designed, and complex functionalities such as angle-multiplexed metasurface holograms are also demonstrated. However, the construction of the CMT model involves conventional design approaches of metalenses. Moreover, an initial dielectric distribution is required.

In contrast, the global population-based optimizers reframe the optimization process as the training of a generative neural network^190^. Researchers in Fan’s group have given several examples^192,193^. Let’s take a global optimizer presented by Jiang et al.^193^ for example. Instead of a training set of known devices, the global topology optimization networks (GLOnets) learn the physical relationship between device geometry and response directly through electromagnetic simulations. As illustrated in Fig. 18d, the conditional GLOnet consists of trainable fully connected, and deconvolutional neural layers. With the input comprised of the desired parameters (λ and output angle *θ*) and random noise vector *z*, the spatial distribution of meta-atoms is the output. During each iteration of training, a batch of devices is generated, and the efficiency gradients *g* between forward and adjoint electromagnetic simulations is then calculated to evaluate the loss function of the network and update the weights of the neurons. As a proof of concept, the GLOnet outputs ensembles of a highly efficient topology-optimized metagrating operating across different wavelengths (600, 900, and 1200 nm) and deflection angles (40°, 60°, and 80°).

### Large-area metalenses

Large-scale and low-cost mass production of practical imaging devices such as eyeglasses for AR/VR/mixed reality (MR) and 3D displays^62,89,194,195^ requires that the designed metalenses have the possibility of large area design and fabrication. In recent years, more and more millimeter-scale metalenses have been experimentally demonstrated^29,64–66,120,194–198^. However, the diameters of metalenses are generally difficult to reach centimeter-scale due to the extremely high data density (owing to millions or billions of individual subwavelength meta-atoms) and the difficulty of mass manufacturing (requiring simple fabrication methods).

#### Design challenges

To reduce the phase error resulting from the coupling of adjacent meta-atoms after the implementation process (step (iii) in the section “Principle”), global simulation and optimization are necessary. Nevertheless, the simulation time conventionally scales approximately as O (R^2.4^) for electromagnetic solvers that utilize standard matrix multiplication (e.g., FDTD method)^199^. Hence, extremely large run-time and high memory space are required during the numerical simulations and optimizations of metalenses on a scale of over millimeters. To avoid running over the computing resource limit, the numerical simulations of metalenses scaled down to micrometer dimension are usually performed instead (Table 2). The scaling trend O (R^2.4^) is also valid for topology-optimized metalenses. To overcome this, Phan et al.^187^ introduced a conceptually new approach for optimizing large-area metasurfaces in a computationally efficient manner. By stitching together individually 3λ-wide optimized sections of the metalens, the computational complexity of total optimization is reduced from high-polynomial to linear. Nevertheless, the diameter of topology-optimized metalenses is rarely exceeding a millimeter size so far. To alleviate these issues, extending computation resources with hardware-accelerated electromagnetic solvers may be another scenario. Hughes et al.^200^ demonstrated a GPU-based hardware-accelerated FDTD solver that enables a full-wave 3D simulation with a size of $100\;{\uplambda} \times 100\;{\uplambda} \times 46\;{\uplambda}$ (66 × 66 × 30.36 μm^3^) in under 5 min. Developing such techniques put simulation of metalenses of the millimeter- and centimeter-scale within reach.

**Table 2:** Methodologies for production of metalens in diameter over 4 mm

Fabrication methodology		Minimal size; height	Metalens diameter		Operating wavelength of the metalens	Constituent/substrate	Ref.
			Sim.	Exp.			
Electron-beam lithography		170 nm; 695 nm	100 μm	1 cm	633 nm (VIS)	Si_3_N_4_/SiO_2_	^46^
		100 nm; 2 μm	–	1 cm	447, 532, 660 nm (VIS)	Exposed resist/glass	^227^
		105 nm; 400 nm	60 μm	5 mm	532 nm (VIS)	Si_3_N_4_/SiO_2_	^194^
Photolithography	193-nm immersion	150 nm; 400 nm	20 μm	8 mm	940 nm (NIR)	a-Si/SiO_2_	^208^
	365-nm stepper	830 nm; 600 nm	100 μm	2 cm	1550 nm (NIR)	a-Si/SiO_2_	^54^
	365-nm stepper	810 nm; 950 nm	–	6 mm	1550 nm (NIR)	a-Si//SWCNT	^53^
	DUV stepper	500 nm; 2 μm	–	1 cm	1550 nm (NIR)	SiN/Si	^131^
	DUV stepper	250 nm; 2 μm	50 μm	1 cm	633 nm (VIS)	SiO_2_/SiO_2_	^147^
	248-nm stepper (LDW-fabricated reticle)	250 nm; 2 μm	500 μm	1 cm	633 nm (VIS)	SiO_2_/SiO_2_	^204^
	UV LDW	2 μm; 10 μm	–	1 cm	10.6 μm (MIR)	Si/Si	^127^
	UV projection (LDW-fabricated reticle)	1.5 μm; 6.8 μm	–	1.2 cm	10.6 μm (MIR)	Si/Si	^228^
Nanoimprint lithography	EBL-fabricated master mold	60 nm; 100 nm	–	2 cm	473, 532, 660 nm (VIS)	Poly-Si/SiO_2_	^62^
	EBL-fabricated master mold	260 nm; 1.2 μm	–	4 mm	940 nm (NIR)	a-Si/Si	^210^
	EBL-fabricated master mold	57 nm; 478 nm	300 μm	4 mm	550 nm (VIS)	TiO_2_/SiO_2_	^211^

#### Demand for minimizing the layout file

A key to the efficient fabrication of metalens is the corresponding layout files. They must undergo computationally demanding processing, such as fracturing, to convert the data into the proper format for manufacturing a reticle. However, unmanageably large layout file sizes are generated for metalenses with diameters over centimeters. For example, the general design file size of a 5-cm-diameter metalens comprised of over 6 billion nanoscale meta-atoms is over 200 gigabytes^54^. To reduce the file size, Capasso’s group proposed a scalable metasurface layout compression algorithm, coined METAC. A library of self-referenced structures is generated by using several layers to represent increasingly doubled copies of a primitive structure. At each radial position, the core algorithm then efficiently assembles appropriate library elements to create the desired structure, forming a ring. The design file size is efficiently reduced to approximately 131 megabytes, which is compressed by 3 orders of magnitude^54^. However, the compression algorithm is valid only for radially symmetric designs. Colburn et al. developed a more general algorithm that does not require any symmetry in the layout^131^. By using a dictionary data structure containing 6 unique nanopost radii as keys, more than a 2600× reduction in memory is achieved for the metasurface in a 1 cm × 1 cm aperture.

#### Scaling up and manufacturing

Another nontrivial challenge posed by large-area metalens is to reach high performance using simple fabrication methods. The performance of metalenses is degraded by a series of fabrication imperfections. For the sake of high resolution, most metalenses especially those operating in the visible regime have been fabricated by electron-beam lithography (EBL) or focused ion beam (FIB) techniques^201^ (including those with millimeter-diameter^35,41,47,65,66,70,71,89,122,128,130,163,202^). Nevertheless, a typical EBL or FIB process to define large area structures suffers from time-consuming and high costs. And the costs explosively increase with the resolution as well as the size. An approach to work out the problem is using projection photolithography which is commonly used in semiconductor foundries. Although customized reticles should be firstly fabricated with UV laser direct writing (LDW)^203^, EBL, or FIB techniques, the feature sizes of the lens are magnified by several times to accommodate the image reduction in the projection system. Hence, the lower resolution is acceptable.

Mass production of metalenses has been demonstrated by stepper/scanner lithography^53,131,204–208^ and nanoimprint lithography (NIL)^62,209–211^ technologies (Table 2), which are the most promising candidates to move metalenses from lab to fab in the future. For example, 1-cm-diameter all-glass metalenses operating at visible wavelength are manufactured using 248-nm deep-ultraviolet (DUV) projection stepper lithography^204^. The pattern of the metalens is replicated rapidly over the face of the 4-inch wafer by repeatedly exposing and incrementally stepping the wafer position. Consequently, the patterning throughput is as high as thousands of metalenses per hour. Alternative to stepper lithography, NIL transfers the pattern of the mask by direct contacting and resin curing, without relying on semiconductor geometrical limitations and processing capacity. Hence, NIL is cost-effective, high-resolution, and high-throughput. However, the contact mode of NIL involves some concerns about defects, throughput, and template wear, especially for a large-area template. For example, thermal NIL^212^, which is widely used, requires high pressure and temperature that easily cause damage to the template and adhesive layer. UV-NIL^209–211^ is performed at low pressures and room temperature, minimizing magnification and distortion errors. Nevertheless, the difficultly discharged bubbles in the UV curable resist cause defects in the meta-atoms.

To solve these problems, a systematical study on the quantitative relationship between processing errors and device performance should be considered. Besides, evaluation for processing errors and standards to guide the industrial production of metasurfaces need to be estimated.

### Device integration

The packaged miniaturized optical systems are required in consumer applications. For high-performance meta-devices, one may consider the necessary elements, the precise alignment, the stability, etc. A few prototypes of imaging systems combining meta-optics and refractive optics have been developed^119,195^. For instance, a portable packaged full-stoke polarization camera is presented by integrating a metagrating with an off-the-shelf catalog lens and a CMOS imaging sensor^119^. The grating sample is 1.5 mm in diameter, but the size of this prototype is much larger owing to the existing bulk lens and optomechanical mounting. A compact spectrometer in millimeter-scale volume is achieved by side-by-side alignment^70,71^ (Fig. 9c, d). Such a device, however, is not suitable for transparent dielectric metalenses. To further reduce the device size for practical applications, achieving an integrated optical module comprised of metalenses and photosensors is vital.

#### Wafer stacking and packaging

**Fig. 19:**
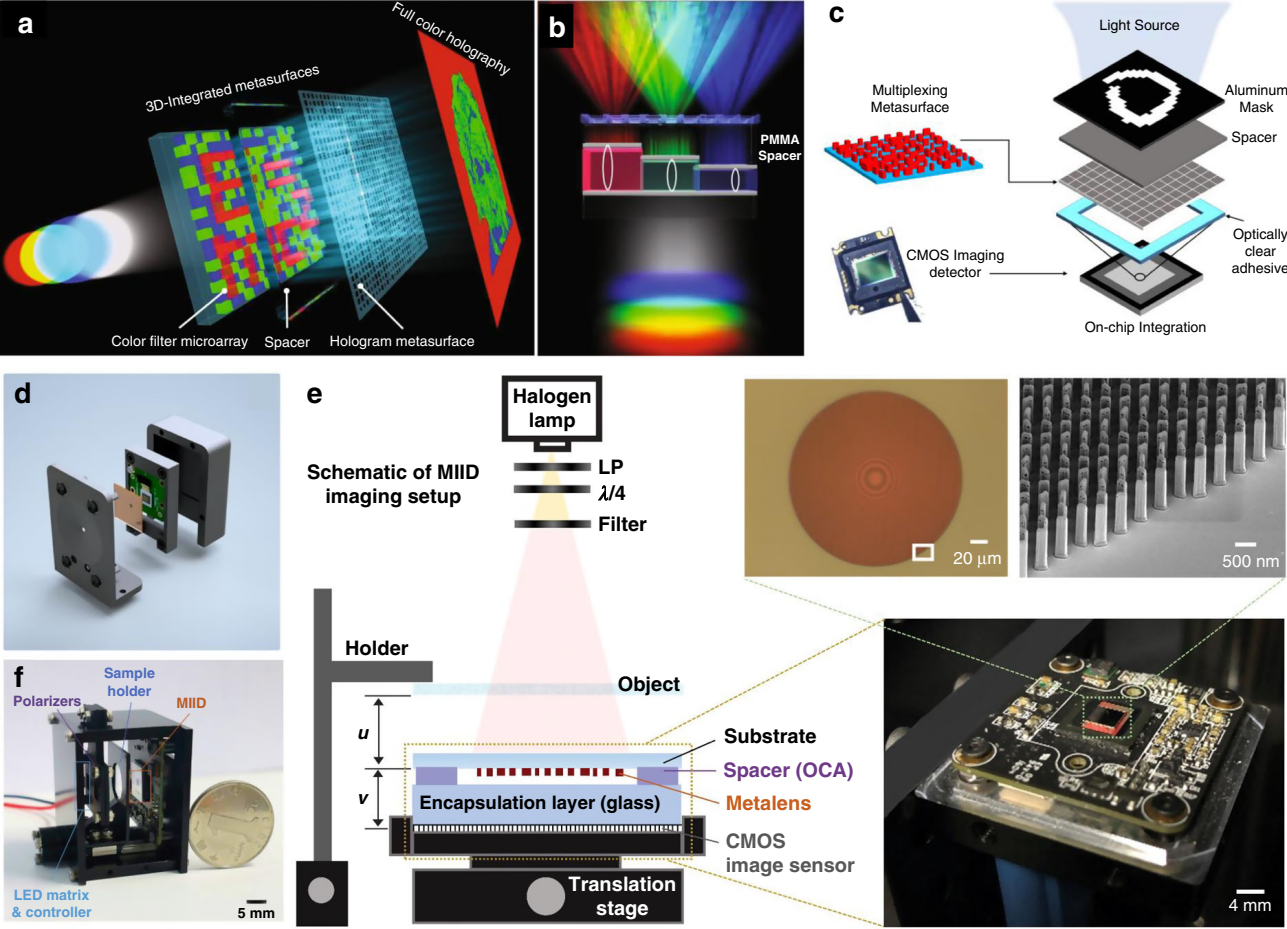
Integration of metasurface and photosensor wafers using stacking and packaging technologies. **a** Exploded view of 3D-integrated metasurfaces for full-color holography. **b** Front view of three micro-units of the 3D-integrated metasurfaces. PMMA is used as a spacer. **c** Exploded schematic diagram of the stacked metasurface integrated with a CMOS chip. **d** Setup of the 3D-printed system integrating the metalens with a photosensor. **e** Schematic of the optical setup for the metalens-integrated imaging device. Zoomed out are the photograph (down), top-view optical microscope image (top left), and side-view SEM image (top right) of the fabricated metalens. **f** Photograph of the prototype. Panels **a** and **b** are reproduced with permission^215^. Copyright 2019, The Authors, published by Springer Nature. Panel **c** is reproduced with permission^216^. Copyright 2022, The Authors, published by Springer Nature. Panel **d** is reproduced with permission^63^. Copyright 2020, American Chemical Society. Panels **e** and **f** are reproduced with permission^60^. Copyright 2020, The Authors. Published by SPIE

The current advanced stacking and packaging technologies^213–218^ offer access to integrating the lens wafer on the photosensor wafer. Hu et al.^215^ demonstrated full-color holography by stacking a metasurface upon a layer of color filter array (Fig. 19a). Considering the stepwise structures for color filters, PMMA is coated as the spacer to form a uniform surface for the metasurface (Fig. 19b). In Hu’s other work^216^, a metasurface-stacked structure is integrated upon a CMOS imaging detector by a layer of optically clear adhesive (OCA) with the desired shape and thickness (Fig. 19c). Martins et al.^63^ proposed a possible prototype of a metalens camera based on a translation stage (Fig. 19d). As shown in Fig. 19e, Xu et al.^60^ mounted the metalens on a CMOS image sensor with OCA tapes. The OCA tapes are also the spacer medium of integration for the well-defined stationary thickness. Hence, the imaging distance *v* is fixed, and a clear image is acquired by tuning the object distance *u* with the translation stage. As a result, high-resolution images (∼1.74 μm) with millimeter-scale image area are achieved by a ∼3-cm size device prototype^60^ (Fig. 19f). Li et al.^61^ demonstrated a similar compact NIR microscopic configuration that gives performances comparable with a commercial microscope.

#### Monolithic integration

The small dimensions of metalenses require fabrication by lithography techniques, where photolithography is currently used as a part of the standard CMOS fabrication process in the microelectronics industry. Hence, a metalens is generally more compatible with a CMOS fabrication line than a conventional lens^2^. In particular, lossless CMOS-compatible materials (e.g., niobium pentoxide and silicon for the visible and infrared wavelengths, respectively)^219^ were chosen to form metalenses, reducing the difficulties of integration preparation and encapsulation. The precise alignment of multiple layers of elements is also achieved^218^. The CMOS compatibility could efficiently help the incorporation of metalenses in the manufacturing process of sensors or other on-chip systems^208,220^.

**Fig. 20:**
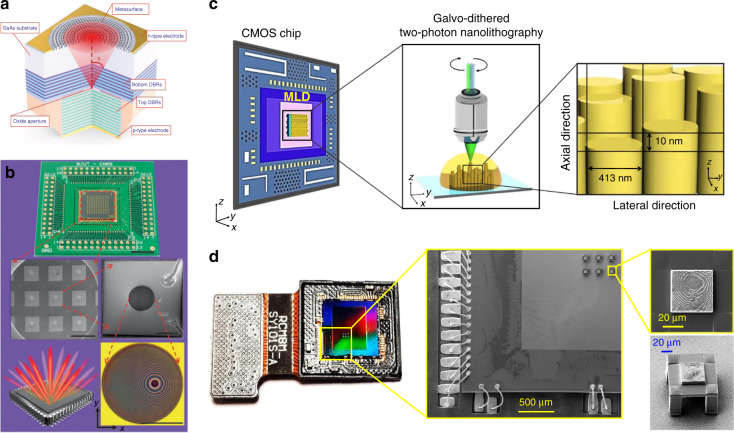
On-chip metasurfaces and the applications. **a** Schematic of the metasurface-VCSELs, depicting the standard VCSEL structure and the beam-shaping metasurface integrated at the backside of the substrate. **b** Schematics of the metasurface -VCSELs chip for wide-range dynamic beam steering applications. **c** Schematic of the metasurface integrated with a CMOS chip through physically 3D printed based on GD-TPN. **d** Photograph of the CMOS sensor with an array of 3 × 2 metasurfaces. Panels **a** and **b** are reproduced with permission^221^. Copyright 2020, The Authors, published by Springer Nature. Panels **c** and **d** are reproduced with permission^222^. Copyright 2021, The Authors, published by Springer Nature

Xie et al.^221^ achieved a monolithic back-emitting configuration for arbitrary beam shaping of vertical-cavity surface-emitting lasers (VCSELs) with wafer-level integration through VCSEL-compatible technology. The VCSEL wafer is fabricated on a GaAs substrate firstly, and then the backside of the substrate (emitting surfaces) is directly sculptured into metasurfaces (Fig. 20a). When the metasurface acts as a focusing lens that compensates for the beam divergence, the emitted light is self-collimated. As a proof of concept, a chip of 10 × 10 metasurface-VCSELs with different deflection angles is fabricated (Fig. 20b).

Goi et al.^222^ manufactured a 3D metasurface on a CMOS image sensor using Galvo-dithered two-photon nanolithography (TPN). Using a dip-in approach and a liquid photoresist in the TPN process (Fig. 20c), an array of 2 × 3 metasurfaces is printed directly on the sensor (Fig. 20d). To precisely regulate the distance between the structures and the imaging plane, the metasurface is mounted on table-like supports with the pillars’ height being 47.1 μm. Similar TPN-based 3D nanoprinting technology is also applied to fabricate metalens on a fiber tip^50^.

#### Other challenges

Encapsulation is necessary for both types of integration. Nevertheless, owing to the index difference between the encapsulation glass and air, the efficiency of the device would be reduced by the reflection at the interface of the two media; the focal length is altered compared with the unpacked metalens, and additional monochromatic aberrations could arise. One also needs to consider the matching of metalens performances and photosensor performances^223^. Hence, the metalens should be designed and optimized according to the overall packaging module from the beginning. For example, the phase profile should be optimized by adding a slab-corrected phase profile^224^.

Moreover, thermal stability and mechanical stability are important in practical applications. The metalens performances would be degraded by variations in refractive index and volume of the constituent material owing to the temperature change. Hence, thermally stable and transparent materials for the operating wavelengths are worth considering. Liu et al.^225^ considered infrared metalenses made of Si and Ge. When the temperature is changed from –40 to 80 °C, the relative variation in focal length is calculated as only 0.032% and 0.121% for 250-μm-diameter metalenses made of Si and Ge, respectively. Nevertheless, the calculation considers the free-standing case. When the metalens is mounted for device integration, the difference between thermal expansion coefficients of the mounting and the metalens materials may cause a surface bending of the metalens, which, in turn, may degrade the focusing performance and damage (crack) the metalenses. Hence, one needs to take the thermal stability and the mechanical stability of metalenses into consideration for metalens-integrated devices.

## Conclusion and outlook

Overall, this review summarizes the progress and challenges of metalenses in miniaturized optical systems. Attributed to the customized engineering capability on the wavefront, the unique features of metalenses, such as aberration-correction, dispersion engineering, and multifunctionality, are utilized in ultra-compact imaging systems. Despite the tremendous progress achieved so far, several challenges should be addressed for the practical development of metalens-based imaging systems mostly aiming at the VIS-NIR-MIR wavelengths. High focusing efficiency is challenging for high-NA metalenses due to the fundamental phase discretization and diffraction constraints. The breakthrough broadband achromatic feature of metalens suffers from some fundamental constraints, design limitations, and fabrication challenges. Moreover, Conflicts in different performances should be manipulated. In addition, common challenges are hindering further development of dielectric metalens-integrated systems, including limitations of conventional design methods, the scaling up of dielectric metalenses, and the approaches for integrated devices.

In turn, these challenges provide some possible future development directions of metalenses. A few examples are given but are not limited to the following. (i) New strategies, both theoretical and experimental, to break the conflicts among metalens performances (efficiency versus NA, NA versus FoV, achromatic bandwidth versus diameter, etc.). (ii) Multifunctional and reconfigurable metalenses that could replace complicated configurations of conventional optics (e.g., polarization cameras, zoom lenses, full-optical setups for signal processing as well as optical analog computing). (iii) High-efficiency design methods for large-area and high-performance metalenses (such as end-to-end intelligent designs, freeform optimizations, as well as computational imaging techniques where the aberration correction is offloaded to post-processing software). (iv) High-efficiency fabrication and mass manufacturing methodologies for large-area metalenses. A systematical model on the quantitative relationship between processing errors and device performance would be helpful, and we expect that the evaluations and standards to guide the industrial production of metasurfaces would be estimated.

Another interesting direction for metalenses (local flat-optics) in compact imaging systems is the combination with nonlocal flat-optics. Even for the simplest imaging system that is composed of a metalens and a photosensor, the free space constitutes a great portion of the system volumes. Recently, the free-space volumes are compressed by a kind of nonlocal flat-optics structure called “spaceplate”, which is characterized by a momentum-dependent transfer function. After transmitting through the spaceplate with a physical length *d*, the output wavefront is equivalent to that has propagated for an equivalent length *d*_eff_ in free space. The space-squeezing effect has been demonstrated using 3D photonic crystals with isotropic band structures^226^, uniaxial birefringent slabs (with the same refractive index as the background for the extraordinary light and a larger index for ordinary light)^78^, and multilayered structures^78,79^. Particularly, the potential of combining metalens and spaceplate has been theoretically demonstrated^79^. By matching the compression ratio (*d*_eff_ /*d*) of the spaceplate and the imaging distance, an ultrathin, fully solid-state imaging system is potentially achieved with a metalens and a photosensor integrated on the two sides of the spaceplate, respectively. However, there remain some issues to be addressed related to combining metalenses and spaceplates. For instance, since the propagating momentum relies on not only the propagating direction but also the wavelength, there would be a trade-off between the achromatic bandwidth and the compression ratio.

By addressing the present challenges of metalenses and combining the nonlocal optics, we envision that metalens-based imaging systems would be increasingly compact and widely employed in future applications, ranging from camera modules for consumer photography and autonomous vehicles to wearable displays for AR/VR/MR and machine vision, to bioimaging and endoscopy, to signal processing and optical computation.

## References

[1] Huang K (2018). Planar diffractive lenses: fundamentals, functionalities, and applications. Adv. Mater..

[2] Engelberg J, Levy U (2020). The advantages of metalenses over diffractive lenses. Nat. Commun..

[3] Kim SJ (2021). Dielectric metalens: properties and three-dimensional imaging applications. Sensors.

[4] Chen WT, Capasso F (2021). Will flat optics appear in everyday life anytime soon?. Appl. Phys. Lett..

[5] Zou XJ (2020). Imaging based on metalenses. PhotoniX.

[6] Chen MK (2021). Principles, functions, and applications of optical meta-lens. Adv. Optical Mater..

[7] Chen WT, Zhu AY, Capasso F (2020). Flat optics with dispersion-engineered metasurfaces. Nat. Rev. Mater..

[8] Lee GY, Sung J, Lee B (2020). Metasurface optics for imaging applications. MRS Bull..

[9] Ee HS, Agarwal R (2016). Tunable metasurface and flat optical zoom lens on a stretchable substrate. Nano Lett..

[10] Genevet P (2017). Recent advances in planar optics: from plasmonic to dielectric metasurfaces. Optica.

[11] Aieta F (2013). Aberrations of flat lenses and aplanatic metasurfaces. Opt. Express.

[12] Ottevaere, H. & Thienpont, H. Optical microlenses. in *Encyclopedia of Modern Optics* (ed Guenther, R. D.) 21–43 (Amsterdam: Elsevier, 2005).

[13] Maréchal A (1947). Mechanical integrator for studying the distribution of light in the optical image. J. Optical Soc. Am..

[14] Wang RX (2021). Metalens for generating a customized vectorial focal curve. Nano Lett..

[15] Fan XH (2020). Axially tailored light field by means of a dielectric metalens. Phys. Rev. Appl..

[16] Chang WH (2021). Generation of concentric space-variant linear polarized light by dielectric metalens. Nano Lett..

[17] Zhou T (2020). Spin-independent metalens for helicity–multiplexing of converged vortices and cylindrical vector beams. Opt. Lett..

[18] Wang W (2020). Polarization multiplexing and bifocal optical vortex metalens. Results Phys..

[19] Hu YF (2021). Dielectric metasurface zone plate for the generation of focusing vortex beams. PhotoniX.

[20] Huang BQ (2020). Multifocal co-plane metalens based on computer-generated holography for multiple visible wavelengths. Results Phys..

[21] Zhang JP (2021). A vortex-focused beam metalens array in the visible light range based on computer-generated holography. Results Phys..

[22] Decker M (2015). High-efficiency dielectric huygens’ surfaces. Adv. Optical Mater..

[23] Shalaev MI (2015). High-efficiency all-dielectric metasurfaces for ultracompact beam manipulation in transmission mode. Nano Lett..

[24] Zhang L (2018). Ultra-thin high-efficiency mid-infrared transmissive huygens meta-optics. Nat. Commun..

[25] Yu YF (2015). High-transmission dielectric metasurface with 2π phase control at visible wavelengths. Laser Photonics Rev..

[26] Yu JB (2021). Dielectric super-absorbing metasurfaces via pt symmetry breaking. Optica.

[27] Tian JY (2020). High-*Q* all-dielectric metasurface: super and suppressed optical absorption. ACS Photonics.

[28] Shi T (2021). Displacement-mediated bound states in the continuum in all-dielectric superlattice metasurfaces. PhotoniX.

[29] Arbabi A (2015). Efficient dielectric metasurface collimating lenses for mid-infrared quantum cascade lasers. Opt. Express.

[30] Anzan-Uz-Zaman M (2020). A novel approach to Fabry–Pérot-resonance-based lens and demonstrating deep-subwavelength imaging. Sci. Rep..

[31] Li J (2019). Mechanisms of 2π phase control in dielectric metasurface and transmission enhancement effect. Opt. Express.

[32] Chen C (2021). Metasurfaces with planar chiral meta-atoms for spin light manipulation. Nano Lett..

[33] Pan MY (2018). Circular-polarization-sensitive absorption in refractory metamaterials composed of molybdenum zigzag arrays. Opt. Express.

[34] Chen BH (2017). Gan metalens for pixel-level full-color routing at visible light. Nano Lett..

[35] Khorasaninejad M (2016). Polarization-insensitive metalenses at visible wavelengths. Nano Lett..

[36] Khorasaninejad M, Capasso F (2015). Broadband multifunctional efficient meta-gratings based on dielectric waveguide phase shifters. Nano Lett..

[37] Chen R (2020). Multifunctional metasurface: coplanar embedded design for metalens and nanoprinted display. ACS Photonics.

[38] Arbabi A (2015). Dielectric metasurfaces for complete control of phase and polarization with subwavelength spatial resolution and high transmission. Nat. Nanotechnol..

[39] Pancharatnam S (1956). Generalized theory of interference and its applications. Proc. Indian Acad. Sci. – Sect. A.

[40] Berry MV (1984). Quantal phase factors accompanying adiabatic changes. Proc. R. Soc. A: Math., Phys. Eng. Sci..

[41] Khorasaninejad M (2016). Metalenses at visible wavelengths: diffraction-limited focusing and subwavelength resolution imaging. Science.

[42] Mueller JPB (2017). Metasurface polarization optics: independent phase control of arbitrary orthogonal states of polarization. Phys. Rev. Lett..

[43] Li SQ (2019). Multidimensional manipulation of photonic spin hall effect with a single-layer dielectric metasurface. Adv. Optical Mater..

[44] Yuan YY (2020). A fully phase-modulated metasurface as an energy-controllable circular polarization router. Adv. Sci..

[45] Chen WT (2017). Immersion meta-lenses at visible wavelengths for nanoscale imaging. Nano Lett..

[46] Fan ZB (2018). Silicon nitride metalenses for close-to-one numerical aperture and wide-angle visible imaging. Phys. Rev. Appl..

[47] Liang HW (2018). Ultrahigh numerical aperture metalens at visible wavelengths. Nano Lett..

[48] Huang TY (2019). A monolithic immersion metalens for imaging solid-state quantum emitters. Nat. Commun..

[49] Paniagua-Domínguez R (2018). A metalens with a near-unity numerical aperture. Nano Lett..

[50] Hadibrata W (2021). Inverse design and 3D printing of a metalens on an optical fiber tip for direct laser lithography. Nano Lett..

[51] Chantakit T (2020). All-dielectric silicon metalens for two-dimensional particle manipulation in optical tweezers. Photonics Res..

[52] Plidschun M (2021). Ultrahigh numerical aperture meta-fibre for flexible optical trapping. Light Sci. Appl..

[53] She A (2018). Adaptive metalenses with simultaneous electrical control of focal length, astigmatism, and shift. Sci. Adv..

[54] She A (2018). Large area metalenses: design, characterization, and mass manufacturing. Opt. Express.

[55] Wang YJ (2021). High-efficiency broadband achromatic metalens for near-IR biological imaging window. Nat. Commun..

[56] Sell D (2017). Periodic dielectric metasurfaces with high-efficiency, multiwavelength functionalities. Adv. Optical Mater..

[57] Arbabi A (2020). Increasing efficiency of high numerical aperture metasurfaces using the grating averaging technique. Sci. Rep..

[58] Byrnes SJ (2016). Designing large, high-efficiency, high-numerical-aperture, transmissive meta-lenses for visible light. Opt. Express.

[59] Kalvach A, Szabó Z (2016). Aberration-free flat lens design for a wide range of incident angles. J. Optical Soc. Am. B.

[60] Xu BB (2020). Metalens-integrated compact imaging devices for wide-field microscopy. Adv. Photonics.

[61] Li ZX (2021). Compact metalens-based integrated imaging devices for near-infrared microscopy. Opt. Express.

[62] Lee GY (2018). Metasurface eyepiece for augmented reality. Nat. Commun..

[63] Martins A (2020). On metalenses with arbitrarily wide field of view. ACS Photonics.

[64] Hao CL (2020). Single-layer aberration-compensated flat lens for robust wide-angle imaging. Laser Photonics Rev..

[65] Engelberg J (2020). Near-IR wide-field-of-view huygens metalens for outdoor imaging applications. Nanophotonics.

[66] Shalaginov MY (2020). Single-element diffraction-limited fisheye metalens. Nano Lett..

[67] Groever B, Chen WT, Capasso F (2017). Meta-lens doublet in the visible region. Nano Lett..

[68] Kim C, Kim SJ, Lee B (2020). Doublet metalens design for high numerical aperture and simultaneous correction of chromatic and monochromatic aberrations. Opt. Express.

[69] Arbabi A (2016). Miniature optical planar camera based on a wide-angle metasurface doublet corrected for monochromatic aberrations. Nat. Commun..

[70] Faraji-Dana M (2018). Compact folded metasurface spectrometer. Nat. Commun..

[71] Faraji-Dana M (2019). Hyperspectral imager with folded metasurface optics. ACS Photonics.

[72] Liang HW (2019). High performance metalenses: numerical aperture, aberrations, chromaticity, and trade-offs. Optica.

[73] Fan CY, Lin CP, Su GDJ (2020). Ultrawide-angle and high-efficiency metalens in hexagonal arrangement. Sci. Rep..

[74] Zhang Q (2020). High-numerical-aperture dielectric metalens for super-resolution focusing of oblique incident light. Adv. Optical Mater..

[75] Luo XG (2021). Recent advances of wide-angle metalenses: principle, design, and applications. Nanophotonics.

[76] Qiu M (2018). Angular dispersions in terahertz metasurfaces: physics and applications. Phys. Rev. Appl..

[77] Zhang XY (2020). Controlling angular dispersions in optical metasurfaces. Light Sci. Appl..

[78] Reshef O (2021). An optic to replace space and its application towards ultra-thin imaging systems. Nat. Commun..

[79] Chen AB, Monticone F (2021). Dielectric nonlocal metasurfaces for fully solid-state ultrathin optical systems. ACS Photonics.

[80] Shi ZJ (2020). Continuous angle-tunable birefringence with freeform metasurfaces for arbitrary polarization conversion. Sci. Adv..

[81] Arbabi E (2016). Multiwavelength polarization-insensitive lenses based on dielectric metasurfaces with meta-molecules. Optica.

[82] Avayu O (2017). Composite functional metasurfaces for multispectral achromatic optics. Nat. Commun..

[83] Tang F (2020). Dielectric metalenses at long-wave infrared wavelengths: multiplexing and spectroscope. Results Phys..

[84] Arbabi E (2017). Controlling the sign of chromatic dispersion in diffractive optics with dielectric metasurfaces. Optica.

[85] Chen WT (2018). A broadband achromatic metalens for focusing and imaging in the visible. Nat. Nanotechnol..

[86] Chen C (2019). Spectral tomographic imaging with aplanatic metalens. Light Sci. Appl..

[87] Pahlevaninezhad H (2018). Nano-optic endoscope for high-resolution optical coherence tomography in vivo. Nat. Photonics.

[88] Wang SM (2018). A broadband achromatic metalens in the visible. Nat. Nanotechnol..

[89] Li ZY (2021). Meta-optics achieves RGB-achromatic focusing for virtual reality. Sci. Adv..

[90] Shrestha S (2018). Broadband achromatic dielectric metalenses. Light Sci. Appl..

[91] Feng X (2021). Optical multiparameter detection system based on a broadband achromatic metalens array. Adv. Optical Mater..

[92] Zhou HP (2019). Broadband achromatic metalens in the midinfrared range. Phys. Rev. Appl..

[93] Ou K (2021). Broadband achromatic metalens in mid-wavelength infrared. Laser Photonics Rev..

[94] Li XS (2021). Transmissive mid-infrared achromatic bifocal metalens with polarization sensitivity. Opt. Express.

[95] Zhang SY (2018). Solid-immersion metalenses for infrared focal plane arrays. Appl. Phys. Lett..

[96] Song NT (2021). Broadband achromatic metasurfaces for longwave infrared applications. Nanomaterials.

[97] Khorasaninejad M (2017). Achromatic metalens over 60 nm bandwidth in the visible and metalens with reverse chromatic dispersion. Nano Lett..

[98] Hu, Y. Q. et al. Ultra-broadband dispersion-manipulated dielectric metalenses by nonlinear dispersive phase compensation. Preprint at https://arxiv.org/abs/2112.14127 (2021).

[99] Fan ZB (2019). A broadband achromatic metalens array for integral imaging in the visible. Light Sci. Appl..

[100] Lin RJ (2019). Achromatic metalens array for full-colour light-field imaging. Nat. Nanotechnol..

[101] Presutti F, Monticone F (2020). Focusing on bandwidth: achromatic metalens limits. Optica.

[102] Mann SA, Sounas DL, Alù A (2019). Nonreciprocal cavities and the time–bandwidth limit. Optica.

[103] Zhang F (2018). Metasurfaces for broadband dispersion engineering through custom-tailored multi-resonances. Appl. Phys. Express.

[104] Wang SM (2017). Broadband achromatic optical metasurface devices. Nat. Commun..

[105] Monticone F, Alù A (2016). Invisibility exposed: physical bounds on passive cloaking. Optica.

[106] Tucker RS, Ku PC, Chang-Hasnain CJ (2005). Slow-light optical buffers: capabilities and fundamental limitations. J. Lightwave Technol..

[107] Miller DAB (2007). Fundamental limit to linear one-dimensional slow light structures. Phys. Rev. Lett..

[108] Yin X (2019). Hyperbolic metamaterial devices for wavefront manipulation. Laser Photonics Rev..

[109] Aieta F (2015). Multiwavelength achromatic metasurfaces by dispersive phase compensation. Science.

[110] Balli F (2020). A hybrid achromatic metalens. Nat. Commun..

[111] Li MM (2020). Dual-layer achromatic metalens design with an effective abbe number. Opt. Express.

[112] Balli F (2021). An ultrabroadband 3D achromatic metalens. Nanophotonics.

[113] Mansouree M (2020). Multifunctional 2.5D metastructures enabled by adjoint optimization. Optica.

[114] Dai XM (2021). Holographic super-resolution metalens for achromatic sub-wavelength focusing. ACS Photonics.

[115] Hu YQ (2020). All-dielectric metasurfaces for polarization manipulation: principles and emerging applications. Nanophotonics.

[116] Khorasaninejad M, Capasso F (2017). Metalenses: versatile multifunctional photonic components. Science.

[117] Sattar, S. et al. Review of spectral and polarization imaging systems. *Proceedings of SPIE 11351, Unconventional Optical Imaging II. SPIE, 113511Q* (SPIE, 2020).

[118] Gruev V, Perkins R, York T (2010). CCD polarization imaging sensor with aluminum nanowire optical filters. Opt. Express.

[119] Rubin NA (2019). Matrix fourier optics enables a compact full-stokes polarization camera. Science.

[120] Zhao F (2021). Metalens-assisted system for underwater imaging. Laser Photonics Rev..

[121] Zhang XQ (2019). Direct polarization measurement using a multiplexed Pancharatnam-Berry metahologram. Optica.

[122] Khorasaninejad M (2016). Multispectral chiral imaging with a metalens. Nano Lett..

[123] Tang LL (2020). Spin-dependent dual-wavelength multiplexing metalens. Opt. Lett..

[124] Yang ZY (2018). Generalized Hartmann-Shack array of dielectric metalens sub-arrays for polarimetric beam profiling. Nat. Commun..

[125] Arbabi E (2018). Full-stokes imaging polarimetry using dielectric metasurfaces. ACS Photonics.

[126] Chen C (2020). Parallel polarization illumination with a multifocal axicon metalens for improved polarization imaging. Nano Lett..

[127] Yan C (2019). Midinfrared real-time polarization imaging with all-dielectric metasurfaces. Appl. Phys. Lett..

[128] Wei YX (2020). Compact optical polarization-insensitive zoom metalens doublet. Adv. Optical Mater..

[129] Han ZY (2020). MEMS-actuated metasurface alvarez lens. Microsyst. Nanoengineering.

[130] Luo Y (2021). Varifocal metalens for optical sectioning fluorescence microscopy. Nano Lett..

[131] Colburn S, Zhan A, Majumdar A (2018). Varifocal zoom imaging with large area focal length adjustable metalenses. Optica.

[132] Iwami K (2020). Demonstration of focal length tuning by rotational varifocal moiré metalens in an ir-A wavelength. Opt. Express.

[133] Arbabi E (2018). MEMS-tunable dielectric metasurface lens. Nat. Commun..

[134] Kamali SM (2016). Highly tunable elastic dielectric metasurface lenses. Laser Phontonics Rev..

[135] Wei SB (2021). A varifocal graphene metalens for broadband zoom imaging covering the entire visible region. ACS Nano.

[136] Fu R (2019). Reconfigurable step-zoom metalens without optical and mechanical compensations. Opt. Express.

[137] Lin RH, Li XH (2019). Multifocal metalens based on multilayer Pancharatnam-Berry phase elements architecture. Opt. Lett..

[138] Tian SN (2019). Dielectric longitudinal bifocal metalens with adjustable intensity and high focusing efficiency. Opt. Express.

[139] Gao S (2019). Twofold polarization-selective all-dielectric trifoci metalens for linearly polarized visible light. Adv. Optical Mater..

[140] Aiello MD (2019). Achromatic varifocal metalens for the visible spectrum. ACS Photonics.

[141] Yao Z, Chen YH (2021). Focusing and imaging of a polarization-controlled bifocal metalens. Opt. Express.

[142] Li L (2021). Broadband polarization-switchable multi-focal noninterleaved metalenses in the visible. Laser Photonics Rev..

[143] Chen XZ (2015). Longitudinal multifoci metalens for circularly polarized light. Adv. Optical Mater..

[144] Abdollahramezani, S. et al. Reconfigurable multifunctional metasurfaces employing hybrid phase-change plasmonic architecture. Preprint at https://arxiv.org/abs/1809.08907 (2018). 10.1515/nanoph-2022-0271 PMC11501198 39635187

[145] Yin XH (2017). Beam switching and bifocal zoom lensing using active plasmonic metasurfaces. Light Sci. Appl..

[146] Shalaginov MY (2021). Reconfigurable all-dielectric metalens with diffraction-limited performance. Nat. Commun..

[147] Lininger A (2020). Optical properties of metasurfaces infiltrated with liquid crystals. Proc. Natl Acad. Sci. USA.

[148] Hu YQ (2021). Electrically tunable multifunctional polarization-dependent metasurfaces integrated with liquid crystals in the visible region. Nano Lett..

[149] Zhou SH (2020). Liquid crystal integrated metalens with dynamic focusing property. Opt. Lett..

[150] Fan CY (2020). Electrically modulated varifocal metalens combined with twisted nematic liquid crystals. Opt. Express.

[151] Badloe T (2021). Electrically tunable bifocal metalens with diffraction-limited focusing and imaging at visible wavelengths. Adv. Sci..

[152] Klopfer E (2020). Dynamic focusing with high-quality-factor metalenses. Nano Lett..

[153] Berto P (2019). Tunable and free-form planar optics. Nat. Photonics.

[154] Afridi A (2018). Electrically driven varifocal silicon metalens. ACS Photonics.

[155] Hu JT (2019). Lattice-resonance metalenses for fully reconfigurable imaging. ACS Nano.

[156] Shalaginov MY (2020). Design for quality: reconfigurable flat optics based on active metasurfaces. Nanophotonics.

[157] Xu ZQ (2020). Spatially resolved dynamically reconfigurable multilevel control of thermal emission. Laser Photonics Rev..

[158] Pan MY (2020). Multi-band middle-infrared-compatible camouflage with thermal management via simple photonic structures. Nano Energy.

[159] Wang YF (2021). Electrical tuning of phase-change antennas and metasurfaces. Nat. Nanotechnol..

[160] Xu ZQ (2021). Nonvolatile optically reconfigurable radiative metasurface with visible tunability for anticounterfeiting. Nano Lett..

[161] Liu WW (2020). Aberration-corrected three-dimensional positioning with a single-shot metalens array. Optica.

[162] Holsteen AL (2019). A light-field metasurface for high-resolution single-particle tracking. Nano Lett..

[163] Guo Q (2019). Compact single-shot metalens depth sensors inspired by eyes of jumping spiders. Proc. Natl Acad. Sci. USA.

[164] Park MK (2020). Virtual-moving metalens array enabling light-field imaging with enhanced resolution. Adv. Optical Mater..

[165] Jin CQ (2019). Dielectric metasurfaces for distance measurements and three-dimensional imaging. Adv. Photonics.

[166] Colburn S, Majumdar A (2020). Metasurface generation of paired accelerating and rotating optical beams for passive ranging and scene reconstruction. ACS Photonics.

[167] Li XY (2020). Graphene metalens for particle nanotracking. Photonics Res..

[168] Zhou HQ (2020). All-dielectric bifocal isotropic metalens for a single-shot hologram generation device. Opt. Express.

[169] Abdollahramezani S, Hemmatyar O, Adibi A (2020). Meta-optics for spatial optical analog computing. Nanophotonics.

[170] Kwon H (2020). Single-shot quantitative phase gradient microscopy using a system of multifunctional metasurfaces. Nat. Photonics.

[171] Zhao MX (2021). Phase characterisation of metalenses. Light Sci. Appl..

[172] Elsawy MMR (2021). Multiobjective statistical learning optimization of RGB metalens. ACS Photonics.

[173] Wang N (2021). Intelligent designs in nanophotonics: from optimization towards inverse creation. PhotoniX.

[174] Chen SH (2022). Multi-objective thermo-economic optimization of Collins cycle. Energy.

[175] Jiang L (2021). Neural network enabled metasurface design for phase manipulation. Opt. Express.

[176] An SS (2020). Deep learning modeling approach for metasurfaces with high degrees of freedom. Opt. Express.

[177] Qu YR (2019). Migrating knowledge between physical scenarios based on artificial neural networks. ACS Photonics.

[178] An SS (2019). A deep learning approach for objective-driven all-dielectric metasurface design. ACS Photonics.

[179] Wang FL (2022). Visible achromatic metalens design based on artificial neural network. Adv. Optical Mater..

[180] So S (2020). Deep learning enabled inverse design in nanophotonics. Nanophotonics.

[181] Tseng E (2021). Neural nano-optics for high-quality thin lens imaging. Nat. Commun..

[182] Saigre-Tardif C (2022). Intelligent meta-imagers: from compressed to learned sensing. Appl. Phys. Rev..

[183] del Hougne, P. From compressed sensing to learned sensing with metasurface imagers. *Proceedings of SPIE 11745, Passive and Active Millimeter-Wave Imaging XXIV*. (SPIE, 2021).

[184] del Hougne P (2020). Learned integrated sensing pipeline: reconfigurable metasurface transceivers as trainable physical layer in an artificial neural network. Adv. Sci..

[185] Li HY (2020). Intelligent electromagnetic sensing with learnable data acquisition and processing. Patterns.

[186] Chung H, Miller OD (2020). High-NA achromatic metalenses by inverse design. Opt. Express.

[187] Phan T (2019). High-efficiency, large-area, topology-optimized metasurfaces. Light Sci. Appl..

[188] Lin Z (2021). Computational inverse design for ultra-compact single-piece metalenses free of chromatic and angular aberration. Appl. Phys. Lett..

[189] Park J (2022). Free-form optimization of nanophotonic devices: from classical methods to deep learning. Nanophotonics.

[190] Fan JA (2020). Freeform metasurface design based on topology optimization. MRS Bull..

[191] Zhou M (2021). Inverse design of metasurfaces based on coupled-mode theory and adjoint optimization. ACS Photonics.

[192] Jiang JQ (2019). Free-form diffractive metagrating design based on generative adversarial networks. ACS Nano.

[193] Jiang JQ, Fan JA (2019). Global optimization of dielectric metasurfaces using a physics-driven neural network. Nano Lett..

[194] Wang C (2021). Metalens eyepiece for 3D holographic near-eye display. Nanomaterials.

[195] Nikolov DK (2021). Metaform optics: bridging nanophotonics and freeform optics. Sci. Adv..

[196] Su P (2021). Large-area optical metasurface fabrication using nanostencil lithography. Opt. Lett..

[197] Zhou Y (2020). Flat optics for image differentiation. Nat. Photonics.

[198] Lim SWD, Meretska ML, Capasso F (2021). A high aspect ratio inverse-designed holey metalens. Nano Lett..

[199] Coppersmith D, Winograd S (1990). Matrix multiplication via arithmetic progressions. J. Symb. Comput..

[200] Hughes TW (2021). A perspective on the pathway toward full wave simulation of large area metalenses. Appl. Phys. Lett..

[201] Chen YQ (2021). Sub-10 nm fabrication: methods and applications. Int. J. Extrem. Manuf..

[202] Decker M (2019). Imaging performance of polarization-insensitive metalenses. ACS Photonics.

[203] Geng J (2021). Controllable generation of large-scale highly regular gratings on si films. Light.: Adv. Manuf..

[204] Park JS (2019). All-glass, large metalens at visible wavelength using deep-ultraviolet projection lithography. Nano Lett..

[205] Roy T (2018). Dynamic metasurface lens based on mems technology. APL Photonics.

[206] Zhang SY (2016). High efficiency near diffraction-limited mid-infrared flat lenses based on metasurface reflectarrays. Opt. Express.

[207] Hu T (2020). Cmos-compatible a-Si metalenses on a 12-inch glass wafer for fingerprint imaging. Nanophotonics.

[208] Zhong, Q. Z. et al. Large-area metalens directly patterned on a 12-inch glass wafer using immersion lithography for mass production. *Proceedings of 2020 Optical Fiber Communications Conference and Exhibition*. (San Diego, IEEE, 2020).

[209] Dirdal CA (2020). Towards high-throughput large-area metalens fabrication using uv-nanoimprint lithography and bosch deep reactive ion etching. Opt. Express.

[210] Yoon G (2021). Printable nanocomposite metalens for high-contrast near-infrared imaging. ACS Nano.

[211] Einck VJ (2021). Scalable nanoimprint lithography process for manufacturing visible metasurfaces composed of high aspect ratio TiO_2_ meta-atoms. ACS Photonics.

[212] Pourdavoud N (2017). Photonic nanostructures patterned by thermal nanoimprint directly into organo-metal halide perovskites. Adv. Mater..

[213] Banerji S (2019). Ultra-thin near infrared camera enabled by a flat multi-level diffractive lens. Opt. Lett..

[214] Dannberg P (2014). Wafer-level hybrid integration of complex micro-optical modules. Micromachines.

[215] Hu YQ (2019). 3D-integrated metasurfaces for full-colour holography. Light Sci. Appl..

[216] Luo XH (2022). Metasurface-enabled on-chip multiplexed diffractive neural networks in the visible. Light Sci. Appl..

[217] Cao T (2022). Wideband mid-infrared thermal emitter based on stacked nanocavity metasurfaces. Int. J. Extrem. Manuf..

[218] Luo XH (2020). Integrated metasurfaces with microprints and helicity-multiplexed holograms for real-time optical encryption. Adv. Optical Mater..

[219] Mikheeva E (2020). CMOS-compatible all-dielectric metalens for improving pixel photodetector arrays. APL Photonics.

[220] Li NX (2019). Large-area pixelated metasurface beam deflector on a 12-inch glass wafer for random point generation. Nanophotonics.

[221] Xie YY (2020). Metasurface-integrated vertical cavity surface-emitting lasers for programmable directional lasing emissions. Nat. Nanotechnol..

[222] Goi E (2021). Nanoprinted high-neuron-density optical linear perceptrons performing near-infrared inference on a CMOS chip. Light Sci. Appl..

[223] Miyata M (2021). Full-color-sorting metalenses for high-sensitivity image sensors. Optica.

[224] Groever B (2018). Substrate aberration and correction for meta-lens imaging: an analytical approach. Appl. Opt..

[225] Liu YT (2021). Research progress of aberration analysis and imaging technology based on metalens. Chin. Opt..

[226] Guo C, Wang HW, Fan SH (2020). Squeeze free space with nonlocal flat optics. Optica.

[227] Andrén D (2020). Large-scale metasurfaces made by an exposed resist. ACS Photonics.

[228] Fan QB (2018). A high numerical aperture, polarization-insensitive metalens for long-wavelength infrared imaging. Appl. Phys. Lett..

