# Correction: Dielectric metalens for miniaturized imaging systems: progress and challenges

**DOI:** 10.1038/s41377-022-01041-x

**Published:** 2023-01-04

**Authors:** Meiyan Pan, Yifei Fu, Mengjie Zheng, Hao Chen, Yujia Zang, Huigao Duan, Qiang Li, Min Qiu, Yueqiang Hu

**Affiliations:** 1 Jihua Laboratory, Foshan, 528200 China; 2 College of Mechanical and Vehicle Engineering, Hunan University, Changsha, 410082 China; 3 Greater Bay Area Institute for Innovation, Hunan University, Guangzhou, 511300 Guangdong China; 4 State Key Laboratory of Modern Optical Instrumentation, College of Optical Science and Engineering, Zhejiang University, Hangzhou, 310027 China; 5 Key Laboratory of 3D Micro/Nano Fabrication and Characterization of Zhejiang Province, School of Engineering, Westlake University, 18 Shilongshan Road, Hangzhou, 310024 China; 6 Institute of Advanced Technology, Westlake Institute for Advanced Study, 18 Shilongshan Road, Hangzhou, 310024 China

**Keywords:** Optics and photonics, Physics

Correction to: *Light*: *Science & Applications*

10.1038/s41377-022-00885-7 published online 28 June 2022

After the publication of this article^1^, it is brought to our attention that some mathematical expressions contained errors. Details are listed below.

1. In the paragraph below Eq. (1) on p.3, the expression of the root mean square of WAF contains a typo, which should be: ${{{\mathrm{WAF}}}}_{{{{\mathrm{rms}}}}} = \sqrt{\langle{{{{\mathrm{WAF}}}}\rangle^2 - \langle{{{\mathrm{WAF}}^{{2}}}}\rangle}}$.
2. In the paragraph at the end of p.12, the expression illustrating the relation between time delay and group delay needs to be changed as: ${{\Delta }}T\left( r \right) = GD\left( r \right) - GD(R)$.
3. The correct expression related to the Bode–Fano limit (at the end of p.13) should be: $\Delta \omega _{{{{\mathrm{max}}}}} = - 2\pi c{{{\mathrm{/}}}}[ {{{{\mathrm{log}}}}\left| \Gamma \right|H( {n_{eff}^2 - n_b^2} )}]$.
4. We are correcting Eq. (5) on p. 8, Eq. (7) on p. 12, and Eq. (9) on p. 13, where the refractive index of background (*n*_*b*_) was missing. According to Eq. (1) in the article^1^, the correct form of these equations should be:

5 $$\varphi \left( {r,\lambda ,\theta _i} \right) = - \frac{{2\pi n_b}}{\lambda }\left( {r\,\sin \theta _i + \sqrt {\left( {r - f\tan \theta _i} \right)^2 + f^2} - \frac{f}{{\cos \theta _i}}} \right)$$

7 $$GD\left( r \right) = \frac{{\partial {\upvarphi}\left( {r,\omega } \right)}}{{\partial \omega }} = \frac{{n_b}}{c}\left( {f - \sqrt {r^2 + f^2} } \right)$$

9 $$\Delta \omega \le \Delta \omega _{{{{\mathrm{max}}}}} = \frac{{\kappa c}}{{n_b\left( {\sqrt {f^2 + R^2} - f} \right)}} = \frac{{\kappa c\sqrt {1 - \left( {NA{{{\mathrm{/}}}}n_b} \right)^2} }}{{n_bf\left[ {1 - \sqrt {1 - \left( {NA{{{\mathrm{/}}}}n_b} \right)^2} } \right]}}$$

We would like to apologize for any inconvenience these errors may have caused.

The original article has been updated.
